# Small Molecules Targeting the Structural Dynamics of AR-V7 Partially Disordered Proteins Using Deep Ensemble Docking

**DOI:** 10.1021/acs.jctc.5c00171

**Published:** 2025-04-15

**Authors:** Pantelis Karatzas, Z. Faidon Brotzakis, Haralambos Sarimveis

**Affiliations:** † School of Chemical Engineering, National Technical University of Athens, 9 Heroon Polytechniou Street, Athens 15780, Greece; ‡ Institute of Bioinnovation (IBI), Biomedical Science Research Center Alexander Fleming, 34 Fleming Street, Vari 16672, Greece; § Centre for Misfolding Diseases, Department of Chemistry, University of Cambridge, Lensfield Road, Cambridge CB2 1EW, U.K.

## Abstract

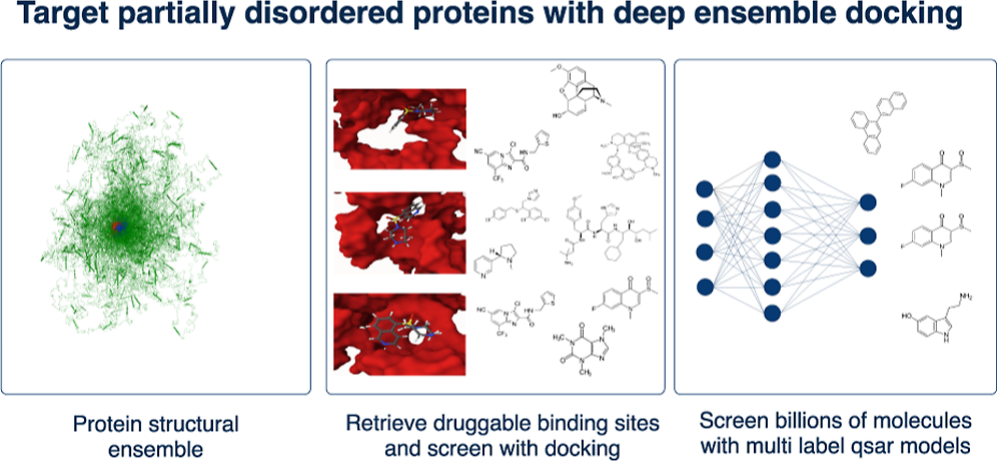

The extensive conformational dynamics of partially disordered proteins hinders the efficiency of traditional in-silico structure-based drug discovery approaches due to the challenge of screening large chemical spaces of compounds, albeit with an excessive number of transient binding sites, quickly making this problem intractable. In this study, using the monomer of the AR-V7 transcription factor splicing variant related to prostate cancer as a test case, we present a deep ensemble docking pipeline that accelerates the screening of small molecule binders targeting partially disordered proteins at functional regions. By swiftly identifying the conformational ensemble of AR-V7 and reducing the dimension of binding sites by a factor of 90, we identify functionally relevant binding sites along the AR-V7 structural ensemble at phase separation-prone regions that have been experimentally shown to contribute to enhanced transcription activity and the onset of tumor growth. Following this, we combine physics-based molecular docking and multiobjective classification machine learning models to speed up the screening for binders in a larger chemical space able to target these functional multiple binding sites of AR-V7. This step increases the multibinding site hit rate of small molecules by a factor of 17 compared to naive molecular docking. Finally, assessing in atomistic molecular dynamics the effect of a selected binder on AR-V7 dynamics, we find that in the presence of the ChEMBL22003 compound, AR-V7 exhibits less conformational entropy, smaller solvent exposure of phase separation-prone regions, and higher solvent exposure of other protein regions, promoting this compound as a potential AR-V7 phase separation modulator.

## 1 Introduction

The process of discovering new drugs is both expensive and time-consuming, facing several hurdles including the challenge of finding effective drug candidates at the preclinical level through high-throughput screening methods, which often have low success rates.^1,2^ To address these issues, computer-aided drug discovery (CADD) has become increasingly important. CADD holds the promise of accelerating and increasing the success rate of drug discovery.^3,4^ A key technique in CADD is molecular docking, an energy-based method that allows quick assessment of the affinity of millions of compounds, such as small molecules and peptides, against various drug targets with a priori known three-dimensional shapes.

However, the application of molecular docking in drug discovery encounters unique challenges when targeting partially disordered proteins (PDPs). PDPs are characterized by a mix of stable folded domains and metastable secondary–tertiary structures and disordered segment regions^1,2,5^ where, in the limiting case of the absence of stable folded domains, these PDPs are also known as intrinsically disordered proteins (IDPs). Due to this interplay, PDPs cannot be characterized by a single conformation alone, thereby rendering conventional drug discovery techniques unsuitable for the design of small molecule drug candidates. In contrast, PDPs can be understood in terms of structural ensembles, which are collections of conformations equipped with underlying population distributions, according to thermodynamics. Such conformations often lack a stable and single active binding site that can be used as a target in CADD screening methods, thus rendering the design of small molecules drug candidates challenging.

Despite these obstacles, identifying binders such as small molecules or peptides targeting PDPs is crucial from both a basic and a drug discovery perspective due to the relevance of PDPs in various biological processes^6^ and their link to a multitude of human diseases such as cancer,^2^ neurodegenerative diseases,^3^ and many other diseases.^6^ Structural analyses of 51 IDPs revealed that PDPs contain, on average, 50% more druggable pockets than fully structured proteins. Moreover, the average probability of finding druggable sites was approximately 9%, compared to just 5% in structured proteins.^7^ Since PDPs encompass both structured and disordered regions and form a broader category than IDPs, it is reasonable to infer that PDPs might similarly exhibit a high potential for druggability. Hence, from the point of view of structure-based drug discovery, it is imperative to have access to structural ensembles of PDPs and to transient binding sites revealed upon their dynamics. Currently, there is no experimental technique available that can directly trace the atomistic structural ensemble of PDPs.^8^ Integrative structural biology methods requiring SAXS or NMR data as input into physics-based molecular dynamics (MD) have greatly assisted in determining structural ensembles of PDPs.^9,10^ AlphaFold has revolutionized protein structure prediction,^11^ with increasing evidence supporting that AlphaFold contains information about conformational dynamics.^12−14^ Despite the increase in efficiency of all-atom and coarse-grained MD force fields of IDPs and PDPs in reproducing experimental data, still the efficiency and accuracy of MD-generated statistical ensembles rely on the accuracy of the force fields, which to the present day remain imperfect. Alternative approaches to generate conformational ensembles such as varying MSA depth^13^ have been proposed to that aim; however, they face challenges in predicting properly weighted statistical ensembles (e.g., following the Boltzmann distribution), thus often predicting conformational ensembles with highly thermodynamically unstable conformations.^15^ An alternative approach to generate structural ensembles of PDPs is AlphaFold Metainference,^12^ a Bayesian inference approach that combines coarse-grained MD with AlphaFold inter-residue distances as restraints, which is able to (a) swiftly generate structural ensembles, (b) introduce into MD structurally meaningful inter-residue distance data from AlphaFold, and (c) achieve that by usage of Metainference, a Bayesian inference approach that quantifies the extent to which a prior distribution of models (e.g., generated by MD) is modified by the introduction of data that are expectation values over a heterogeneous distribution and subject to errors by modeling a finite sample of this distribution, in the spirit of the replica-averaged modeling based on the maximum entropy principle. This approach has been previously shown to increase the efficiency of generated structural ensembles with SAXS data for a variety of IDPs or PDPs against MD or single structure predictions by AlphaFold.

However, even if one has access to the atomistic dynamics of PDPs, from a compound screening perspective, it still remains challenging to determine both a functional yet small number of binding sites that can allow structure-based screening of large libraries for high-affinity molecules against such functional binding sites that can potentially act as modulators of PDP function. The complication lies in the fact that the time scale of brute force structure-based in-silico approaches such as molecular docking calculations scales multiplicatively with the number of binding sites and the number of compounds one attempts to dock. In this study, we present a novel pipeline designed to propose small molecule modulators of PDPs by swiftly generating its structural ensemble by using AlphaFold Metainference, identifying multiple functional binding sites occurring along its dynamics by applying physicochemical and dynamics-based filters, and swiftly screening large libraries of small molecules from ChEMBL against them using multilabel classification deep learning models for high-affinity small molecule binders, which are afterward redocked for validation and subjected to atomistic MD to assess their modulating effect on PDP dynamics.

The proposed methodology is applied to a pertinent case study involving nuclear hormone receptors, such as the androgen receptor (AR) transcription factor, a category of proteins considered largely undruggable.^11^ AR is characterized by a structured ligand-binding domain (LBD), and therapies targeting this domain are commonly used as the first line of treatment for AR-driven prostate cancer.^16,17^ However, approximately 20% of prostate cancer patients advance to a more lethal stage known as castration-resistant prostate cancer (CRPC), the progression of which is often marked by the emergence of constitutively active AR splicing variants such as AR-V7. AR-V7 is devoid of the LBD and consists solely of the DNA-binding domain and an intrinsically disordered activation domain (AD), making them resistant to treatments targeting the LBD.^8,18^ At the molecular level, AR-V7 undergoes phase separation caused by interactions between sticky residues, able to promote its transcriptional activity that leads to tumor growth.^19^ The specific goal of this study is to efficiently identify small molecule modulators of AR-V7 monomer dynamics, able to bind and interfere with sticky and phase separation-prone region binding pockets formed upon AR-V7 monomer dynamics and in so doing can in turn potentially modulate AR-V7 phase separation capacity.

## 2 Methods

### 2.1 Structural Ensemble of AR-V7

In order to generate the structural ensemble, we carried on the protocol described in ref (12). In particular, we start from the sequence of the AR-V7 splicing variant, relevant to prostate cancer.^20^ AR-V7 mRNA retains the first three canonical exons, followed by the variant-specific cryptic exon 3 (CE3). A splicing event at CE3 leads to an LBD-truncated AR-V7. Hence, the sequence of the prostate-relevant AR-V7 splicing variant, henceforth mentioned as AR-V7, corresponds to the activation domain (AD), the DNA binding domain (DBD), and a CE3 domain. By using the AR-V7 sequence, listed in Table S1 and AlphaFold, we predict the structure of AR-V7 and the corresponding AlphaFold predicted distances *d*_*i*,*j*_. AlphaFold-Metainference (AF-MI)^12^ is used to generate the structural ensemble of AR-V7 by combining the physics-based coarse-grained CALVADOS-2^21^ molecular dynamics (MD) model with the AlphaFold-predicted distances, which are used as restraints in the Metainference framework.^22^

#### 2.1.1 AlphaFold-Metainference

Within a Bayesian inference framework, Metainference enables one to determine structural ensembles by coupling prior information and external data coming from experiments or predictions within the maximum entropy principle. Here, we implement Metainference by using the inter-residue distance matrix *d*^AF^ data predicted by AlphaFold, as in ref (12). Metainference disentangles heterogeneous structures from systematic errors (e.g., due to force field or forward model inaccuracies), errors due to the limited sample size of the ensemble, and random errors in the data. Molecular simulations sample from the metainference energy function, *E* = -*k*_B_*T*log(*p*_MI_), where *k*_B_ is the Boltzmann constant, *T* is the temperature, and *p*_MI_ is the metainference, maximum-entropy-compatible, posterior probability distribution
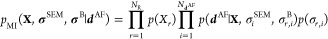
1In this equation, **X** stands for the atomic coordinates vector of the structural ensemble, containing individual replicas *X*_*r*_, (*N*_*R*_ in total); **σ**^SEM^ is the error due to the limited number of replicas in the ensemble; **σ**^B^ is the random and systematic errors in the prior MD force field and in the forward model and the data; and *d*^AF^ is the inter-residue AF distance matrix. **σ**^SEM^ is estimated for each data point (σ_*i*_^SEM^), while **σ**^B^ is calculated per data point *i* and replica *r* as σ_*r*,*i*_^B^. The likelihood *p*(**d̂***AF*|**X**,σ_*i*_^SEM^,σ_*r*,*i*_^B^) takes the form of a Gaussian function
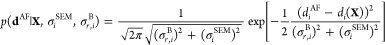
2where *d*_*i*_(*X*) stands for the forward model for data point *i*, i.e., the inter-residue distance between a residue pair in the distance matrix, calculated from the ensemble. The metainference energy function for multiple replicas becomes
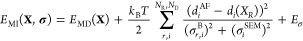
3where *E*_σ_ is the energy term corresponding to all sources of errors
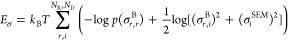
4Finally, *E*_MD_ is the MD force field potential energy function, which here is the CALVADOS-2 force field.^21^ The conformational space *X*_*r*_ is sampled through multireplica simulations (in this study, we used six replicas), and the error parameters for each data point σ_*r*,*i*_^B^ are sampled by Gibbs sampling at each time step. The error sampling range was set to [0.0001,10], and the associated error perturbation in each trial move of the Gibbs sampling was set to 0.1. The error parameter corresponding to the limited number of replicas used to calculate the forward model (σ_*i*_^SEM^) was performed on the fly in a window-averaging fashion every 200 steps of MD. To generate the final unbiased structural ensemble, considering the Parallel Bias Metadynamics weights, we follow the same procedure highlighted in refs (23–25). In particular, we first concatenate the replicas into a concatenated, followed by the usage of the plumed driver to generate the final metadynamics bias per frame by increasing the bias deposition pace to 20,000,000 so that no further bias is added into the trajectory. We then generate the Torrie–Valleau weight of each frame of the concatenated trajectory using the bias per frame. The final ensemble is generated by sampling the concatenated trajectory with these Torrie–Valleau weights.

#### 2.1.2 Distance Selection

However, as in ref (21), we do not consider all AlphaFold inter-residue distances as data to use as restraints but rather a subset. First, since CALVADOS-2 is an IDP-trained coarse-grained model optimized to reproduce structures of disordered regions rather than ordered ones, we use the predicted local distance difference test (pLDDT) score of AlphaFold to define regions with a pLDDT score >0.75 as structured regions. For AR-V7, these regions comprise residues 53–81, 235–244, and 557–628. Such regions are restrained to the AlphaFold-predicted structure by using a root-mean-square deviation (rmsd) potential, and the intraresidue distances corresponding to these structured regions are excluded from the distance restraints.

**Figure 1:**
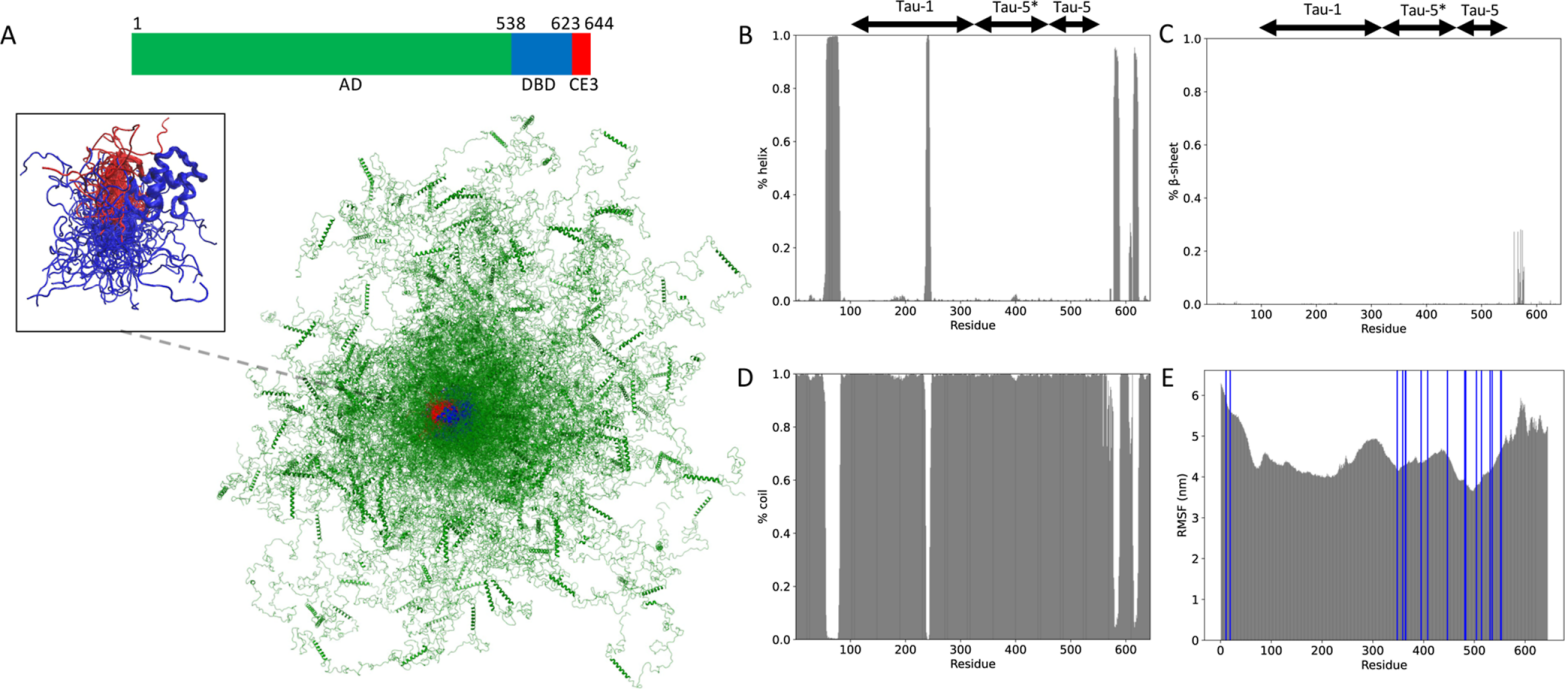
Structural ensemble: (A) primary sequence and structural ensemble of AR-V7. AD, DBD, and CE3 domains are colored in green, blue, and red, respectively. (B,C,D) Residue-based population fraction of α-helix, β-sheet, and coil secondary structure. (E) Residue-based root mean square fluctuation, with blue lines highlighting tyrosine positions that have been characterized by NMR as sticky^32^ and promote phase AR-V7 separation.

For all other inter-residue distances, we consider only residue distances that have a predicted alignment error (PAE) (Figure S1A) lower than 4A and correspond to a total of 2050 inter-residue distances shown in Figure 1D. Interestingly, the AF distance restraints span both short-range interactions (near diagonal) of about 3–12 residues apart (see Figure S1E) as well as longer-range interactions spanning between 50 and 70 residues apart, mostly located around the DNA binding domain (c.a. residues 580–620). We use Metadynamic Metainference^22^ to incorporate he AlphaFold distances as restraints in molecular dynamics. For the MD setup part of AF-MI, we set a 5 fs time step and temperature at 298 K and performed AF-MI simulation in the *NVT* ensemble for 106 steps per replica with a total of six replicas, and frames are saved every 15 ps. To accelerate the sampling, we used a parallel bias metadynamics^26^ potential along four collective variables (Rg1, Rg2, Rg3, and Rg4) representing the radius of gyration of the disordered regions 1–52, 82–234, 245–556, and 629–644, with the following PB-MetaD parameters: Hills height is 0.5 Kj/mol, a deposition pace of 200 MD steps, and a bias factor of 35.

#### 2.1.3 Backmapping

Equivalently to the protocol in ref (12), we then used the PULCHRA software^27^ to backmap to atomistic representations of the structures in the structural ensemble. PULCHRA is a fast and robust method for reconstructing full-atom protein models from simplified or reduced representations, particularly those limited to alpha-carbon atoms. This backmapping process restores the backbone and side-chain atoms of proteins, ensuring correct geometries such as bond lengths and angles. It is based on geometric principles and empirical data derived from known protein structures. It uses knowledge of typical bond lengths, angles, and dihedral angles to restore side-chain and backbone atoms from alpha-carbon coordinates in reduced protein models. This process involves improving local geometry, reconstructing side chains, and correcting the protein’s chirality while ensuring the full-atom model is as close as possible to physically valid configurations. In our protocol, after reconstructing the full atom structure from each coarse-grained structure of the ensemble, we perform energy minimization using the Amber99sb-ildn force field^28^ in vacuum to increase the quality of the atomistic structures. While the backmapping procedure is a difficult process and no perfect reconstruction is mathematically possible, there are many physics and data-driven algorithms that pursue this task, all of them with advantages and pitfalls. We refer the reader to ref (29) for more general information on backmapping algorithms. In this work, we use the PULCHRA algorithm due to its simplicity in implementation. In Figure S3, we report validation statistics of five random structures from our ensemble using MolProbity.^30^ We note the pitfalls of the generated structures in terms of Ramachandran outliers, which are, on average, 10% (PDB threshold 0.05%), poor rotamers 2.34% (PDB threshold 0.3%), and bad angles 3.2% (PDB threshold 0.1%). We, however, note that, on average, these structures contain minor bad bonds and therefore attribute these structures to model predictions rather than structure determination.

### 2.2 Pocket Detection

To detect and analyze binding sites on the structural ensemble of AR-V7, we use pyKVFinder,^31^ an integrable Python package that can swiftly detect cavities using protein PDB files as inputs and characterize them according to different filters such as hydropathy, volume, number of aliphatic apolar, aromatic, polar uncharged, negatively charged, and positively charged amino acids in a cavity. Such physicochemical characterization of the cavities informs the selection of the favorable binding sites for the purpose of in silico molecular docking. For binding site selection, we require that a site contain more than 5 residues and abide by an upper hydropathy filter of 0, a lower/upper area of 80/2480 A^2^, and a lower volume of 120 A^3^ for only the hydrophobic ones that are relatively large to be able to screen relatively large optimizable molecules. Additionally, we apply a third filter, requiring that the root mean squared fluctuation (RMSF) of the residues involved in the cavity should be lower than 4.1 nm^2^. This criterion is motivated by the need for a binding site to be sticky since the aim of this study is to bind sticky residues with small molecules, which in turn aims to inhibit phase separation caused by interactions between sticky residues, as noted in ref (32). This procedure results in the identification of 41 binding sites throughout the ensemble of AR-V7. In Figure S6, we report their structural characteristics such as average size, area, max depth, average depth, and average hydropathy, which are reported as kernel density estimate plots. In Table S2, we report all the residues involved in the detected binding site. These involve tertiary contacts found in the protein structure.

There are multiple ways to perform pocket detection on a pdb. The methodologies can be classified into three categories. They can be based on geometry, on energy, or on evolutionary principles.^33^ pyKVFinder is a geometrical grid-based methodology that can be incorporated in data science pipelines. While there are multiple methodologies available, even ones that combine more than one approach like MegaPocket,^34^ most of these are not easily integrable to data science and data analytics pipelines. We use the reported results from the pyKVFinder to establish the analytical pipeline for cavity selection in Python. For the cavity detection, we did not alter the parameters of the workflow. We included in the analyses the depth and the hydropathy, and as for hydropathy, we use the Eisenberg Weiss scale.^35^ The algorithm has been benchmarked and validated in the published paper.^31^

### 2.3 Molecular Docking

For the screening of the compounds, we randomly sampled a list of nearly 6000 compounds from the ChEMBL database,^36^ for which their logP and molecular weight distributions can be found in Figure S7. We created pdbqt files from the SMILES representation of each molecule using the Ligprep suite and then commenced docking against the selected binding sites. For each of the 41 binding sites of the AR-V7 structural ensemble, we performed molecular docking simulations for each compound using AutoDock Vina, an established open-source software that can screen molecules with relatively good accuracy and bookkeep the lowest binding energy out of five poses.^37^ After completing the docking against the 41 identified binding sites using a machine equipped with 16 CPUs, we compiled the molecules into a single data set containing the SMILES representations and the predicted energy of each molecule for each binding site of the protein. The overall data set consists of 5640 molecules screened against these 41 sites, totaling 231,240 available energies.

### 2.4 Machine Learning

To efficiently screen a vast space of compounds, we utilized the molecular docking data from the previous step to train a QSAR multilabel classification model that predicts the activity of a compound against any of the 41 binding sites. The data set was created as follows: First, we identified the binding energy threshold of the top 5% binding compounds (5% smaller binding energy compounds) for each of the 41 binding sites (see Figures S8–S15). Then, for each binding site, a compound is labeled as active “1” if the docking energy is lower than the energy threshold of that binding site and “0” otherwise. Overall, there are 41 classification end points for each compound. Due to the heavy imbalance toward nonactive molecules, we oversampled the “active” molecules. The model trained on the top 5% of the energies was subsequently used to screen the molecular libraries. The best-performing QSAR multilabel classification model was trained using topological fingerprints as an embedding generated using RDKit and a standard feed-forward neural network. The topological fingerprints were of size of 1412. The QSAR classification model is a feedforward neural network with 4 layers of size 1256. We used the rectified linear unit (ReLU) activation function and added dropout on the first and the final layers to avoid overfitting. In the output layer, we employed a sigmoid function. For loss calculation, we used a binary cross-entropy loss (eq 1), which is typically used in classification and multilabel classification models. The model validation occurred on 10% of the data set, with the training set consisting of 90% of the molecules and the test set comprising the remaining 10% (see Figure S3 for the validation report). We created the descriptors directly from SMILES of the sampled molecules. For model inference, we stored the model with the best overall mean Matthews correlation coefficient (MCC) (eq 2).

The methodology consists of the following steps.

1. Retrieving the Conformational Ensemble: The conformational ensemble was retrieved from the biomolecular target of interest. In this study, we use AlphaFold-MetaInference due to its speed and accuracy efficiency.^12^
2. Pocket Detection and Filtering: The selected binding sites were detected and filtered down. This process can vary between proteins, depending on the biological function of the protein that needs to be altered. In the case of AR-V7, to inhibit aberrant phase separation, which leads to aberrant transcription activation and tumor growth, we aim to detect binding sites around sticky residues as such residues are responsible for the formation of phase separation.
3. Small Molecule Selection and Molecular Docking: A list of small molecules was selected for molecular docking against the detected cavities using randomly sampled molecules from the ChEMBL database, which were then docked in five poses, and the lowest binding energy was bookkept as a label. Other criteria, such as the Lipinski’s Rule of 5, could be used to efficiently subsample the chemical space.
4. QSAR Model Creation and Screening Acceleration: Using the data created in the previous step, a multilabel classification QSAR model was created to accelerate the screening procedure by inferring a vaster chemical space of about 2 million randomly selected compounds from ChEMBL in one cycle of active learning (albeit one can proceed with more consecutive cycles using the QSAR model generated). Specifically, we accept as good predictions the molecules that are predicted as active (“1”) more than 70%, meaning they interact with more than 70% of the docking sites. The threshold can vary from protein to protein and based on screening results.
5. Redocking of Selected Molecules: Molecules passing the threshold are chosen for redocking.

### 2.5 Evaluation Metrics

We evaluated the models for both multiclass classification and per docking site (see Figure S2 for a detailed evaluation report). For the multiclass classification, we used the evaluation metrics of zero–one loss and Hamming loss. For the evaluation of the predictions for each docking site, we used the Matthews correlation coefficient (MCC).

Binary cross-entropy, also known as binary log loss or binary cross-entropy loss, is a commonly used loss function in machine learning. It is primarily used in binary classification problems and is designed to measure the dissimilarity between predicted probability distribution and the true labels of a data set.
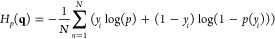
5

The MCC can be quantified as in eq 6, where TP represents the true positives per binding site, TN the true negatives per binding site, FP the false positives per docking site, and FN the false negatives.^38^
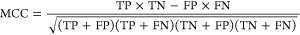
6

The zero–one loss can be quantified as in eq 7, where y are the truth labels of the active/nonactive and *y*^*i*^ are the predicted classes and *n*_samples_ is the number of the samples. In multilabel classification, the zero–one loss corresponds to the subset zero–one loss: for each sample, the entire set of labels must be correctly predicted; otherwise, the loss for that sample is equal to one.^39^
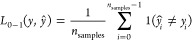
7

The Hamming loss can be quantified as in eq 8, where *y* are the truth labels of the active/nonactive and ŷ are the predicted classes and *n*_samples_ is the number of the samples. The Hamming loss is the fraction of labels that are incorrectly predicted.^39^
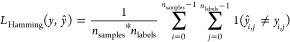
8

### 2.6 Atomistic Parallel Bias Metadynamics Simulations

For the atomistic molecular dynamics of AR-V7 in the presence and absence of ChEMBL22003, reported in the Results and Discussion section “Structural ensemble modulation of AR-V7 by ChEMBL22003”, we initialize our simulations by selecting 33/41 configurations in the AR-V7Metainference structural ensemble, where ChEMBL22003 was found to bind favorably in the redocking calculations. From each of these configurations, we used the Amber99SB-ildn protein force field^28^ and proceeded with two AR-V7 atomistic simulations, one containing ChEMBL22003 bound (holo simulation), which has been parameterized using ACEPYPE,^40^ and another without the compound (apo simulation). For each of these sets of simulations and starting configurations, we perform a protein (plus compound) energy minimization, solvation with 233,447 TIP3P water^41^ molecules, and an 11 NA ion addition to neutralize the charge of the simulation of 100 ps *NPT* where we equilibrate the solvent with backbone position restraints using the Parrinello–Rahman barostat and the velocity rescale thermostat^42,43^ at pressure 1 atm and temperature 310 K, followed by *NVT* of 1 ns. Starting from the last frame of each of these two simulation sets (of 33 simulations each), we performed PB-MetaD using 33 multiple replicas and using four radii of gyration (Rg1, Rg2, Rg3, and Rg4) as collective variables spanning atoms 1–795, 1289–3448, 3599–7854, and 8957–9235, with hill height 0.5 kj/mol, deposition pace 200 steps, and bias factor 35. The aggregate simulation time run along replicas is 115,170 ns. To generate the final unbiased structural ensemble, considering the Parallel Bias Metadynamics weights, we follow the same procedure highlighted in refs (23 and 24). In particular, we first concatenate the replicas into a concatenated trajectory, followed by the usage of the plumed driver to generate the final metadynamics bias per frame by increasing the bias deposition pace to 20,000,000 so that no further bias is added into the trajectory. We then generate the Torrie–Valleau weight of each frame of the concatenated trajectory using the bias per frame. The final ensemble is generated by sampling the concatenated trajectory with these Torrie–Valleau weights. Convergence analysis, in a time-dependent free energy surface calculation per collective variable, is performed in Figure S4.

## 3 Results and Discussion

### 3.1 AR-V7 Structural Ensemble

The structural ensemble of the prostate cancer-relevant AR-V7 splicing variant, generated by AF-MI, is revealed in Figure 1A. AR-V7 exhibits remarkable heterogeneity of structures, especially in correspondence to the AD region of AR-V7, which well captures the current understanding of the IDR part of the AD domain.^19,44^ Unfortunately, due to its intrinsic dynamics, full-length AR-V7 has not been structurally characterized so far in experiments. However, the L26P AR-V7 variant truncated at residues 560–644 (abbreviated WT*) has been characterized by NMR^32^ as per its residue-based helicity. Secondary structure analysis shown in Figure 1B reveals high propensity secondary structure regions (helicity >60%) around residues 56–80 (polyQ domain), 237–244, 578–588, and 614–623 (614SCRLRKCYEAG623). Transient helices (2% < helicity < 10%) form at residues 23–27 (the 23FQNLF27 segment), 173–198, 397–402 (the 397SAWAAA402 segment), and 634–636 (634GNC636). In the NMR measurement of WT*, major helices form at 58–80, 179–183, and 397–403, while transient helices form at 23–271, 232–240, and 351–359. While there is some agreement between our ensemble and the NMR data, it is hard to make a one-to-one comparison due to the difference in sequence of the WT* and the full-length AR-V7 studied here. High beta-sheet propensity shown in Figure 1C is exhibited only at residues 566–577 of the DBD domain, and high propensity of coil regions is manifested in the rest of the sequence as shown in Figure 1D. Since AR-V7 undergoes phase separation, which in turn enhances transcriptional activity,^32^ we embarked on quantifying the sticky residues using as a proxy the residue-based root-mean-square fluctuation quantified by using the structural ensemble. Residues of regions tau1, tau5*, and tau5 exhibit a distinct lower flexibility (rmsf <4.5 nm), pinpointing hindrance and interintramolecular interactions of these regions. Evidently, the tau-5 region contains more sticky residues, in agreement with ^1^H–^15^N NMR^32^ that have identified sticky tyrosine residues (11, 19, 348, 359, 364, 365, 395, 408, 447, 481, 483, 504, 514, 531, 535, 552, and 553) that show a decreased intensity of 1H-15NMR resonance even by more than 50% upon increase of AR-V7 (see Figure 1E) and that upon mutation to Serine in that study inhibit phase separation. We find that 15/17 (80%) of these sticky tyrosine residues have rmsf <4.5 nm in our structural ensemble; hence, we use this extra criterion later to select sticky residue binding pockets (see Methods, Pocket Detection and Results and Discussion, Binding Site Selection).

### 3.2 Binding Site Selection

**Figure 2:**
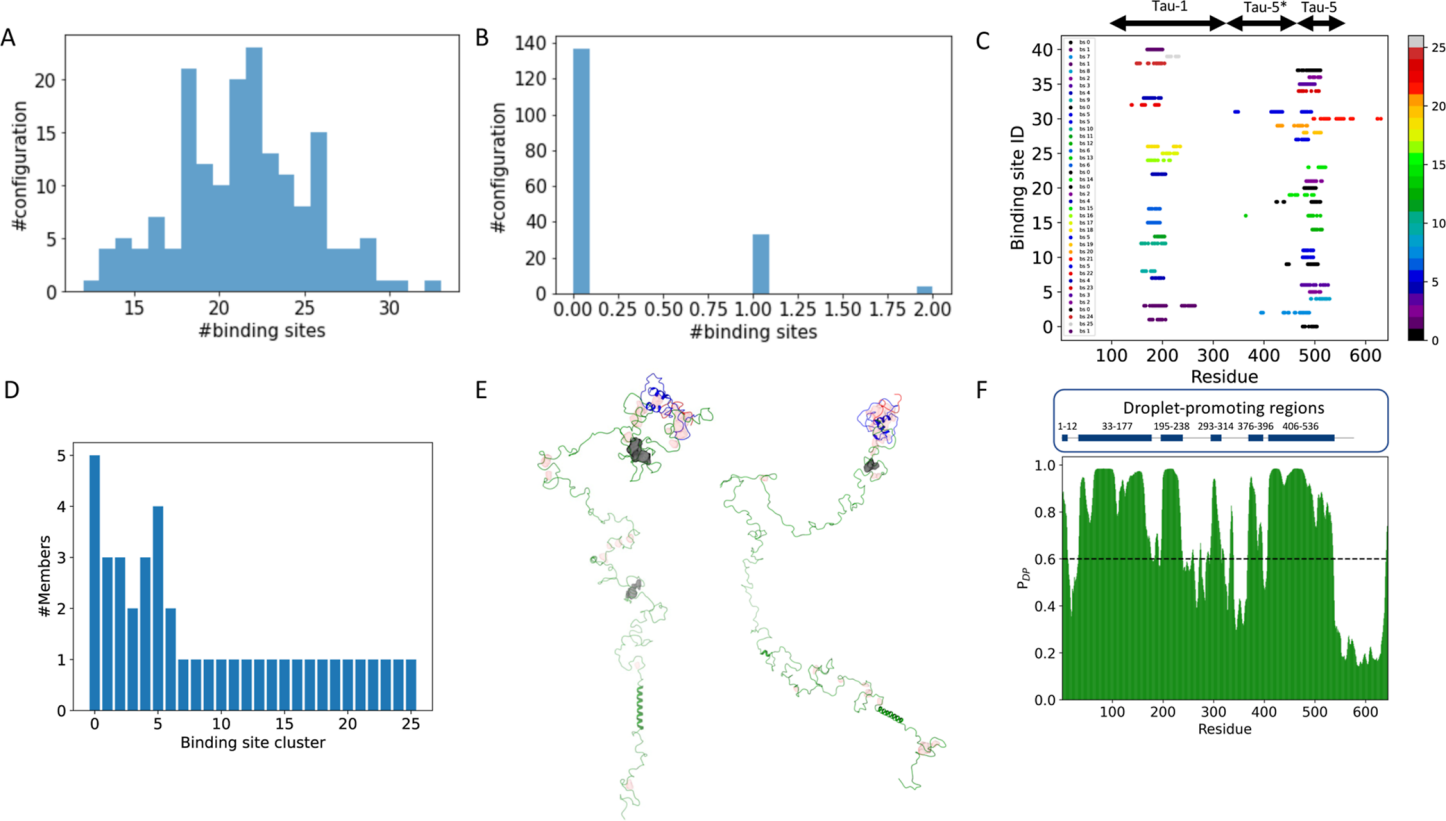
Binding site selection. (A) Distribution of all binding sites throughout the ensemble. (B) Distribution of filtered binding sites throughout the ensemble. (C) Residue-based identity of binding sites. Colors correspond to each binding cluster. (D) Cluster analysis of binding sites. (E) Two example conformations, with red illustrating all binding sites and black the ones that pass the binding site selection filters. The filtered binding sites comprise residues (left) 476–477, 479–81, 488, 493–498, and 501–504 (tau-5 region); (right) 174–179, 181, 190–200, and 206 (tau-1 region); and 394–396, 398, 439–441, 447–449, 460–462, 470–479, and 482–489 (tau-5*/tau-5 region). (F) Residue-based prediction of the probability of droplet formation for AR-V7.

**Figure 3:**
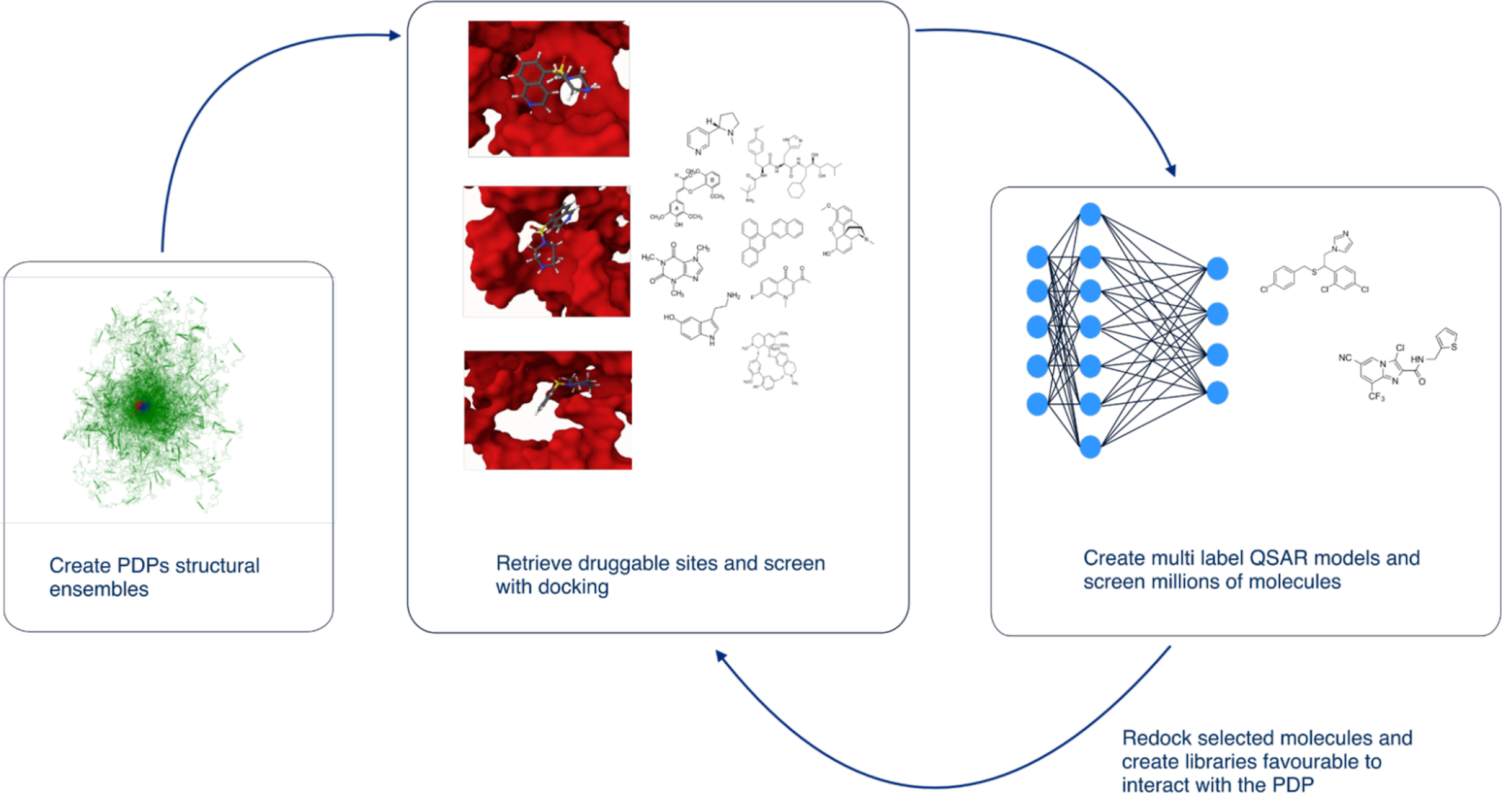
Structural ensemble-based drug discovery using physics and AI models. Multilabel retrieve druggable docking sites from structural ensembles, dock molecules, create multilabel QSAR models, and screen billions of molecules. Selected molecules are redocked and prioritized.

Filtering and selecting the appropriate binding sites are critical in our proposed pipeline. Before filtering, 3718 cavities were detected across the ensemble (Figure 2A), with each conformation in the ensemble displaying multiple binding sites. Molecular docking against all of them is computationally prohibitive. As noted in the Methods section, by focusing on sticky residues and large cavities as quantified by RMSF, hydropathy, and volume/area of cavities, we narrowed down our selection to 41 binding sites (see Figure 2B). This reduction in binding sites was instrumental in decreasing the computational cost of docking calculations by 90-fold. Further analysis of the residue identity of these 41 binding sites is presented in Figure 2C, where we categorize them into 26 distinct binding site groups based on common residue sharing of more than 60%. Binding site clusters 0–6 contain multiple members, while the remaining 19 clusters consist of single members (Figure 2D). Figure 2C shows that most of the binding sites (19 binding sites) are located in subregions of regions tau-5 (residues 450–550), followed by 17 binding sites at tau-1 subregions (residues 150–250) and 5 binding sites (e.g., binding site) in the tau-5*/tau-5 region (residues 350–500), albeit most residues lie in the tau-5 region. Interestingly, most of the binding sites identified in this study are quite novel in the sense that they do not mostly belong in region tau-5*, the region where EPI-001 has been found to bind AR-V7.^32^ In Figure 2E, we show two example configurations where the binding sites are located in the tau-1, tau-5*, and tau-5 regions of the AD domain, corresponding to binding site IDs 0 (spanning tau-5 region), 1 (tau-5 region), and 2 (spanning regions tau-5* and tau-5). More importantly, as shown in Figure 2F, by calculating the LLPS propensity of residues based on the FuzDrop predictor,^45^ we find that the detected pockets in Figure 2C span AR-V7 regions prone to LLPS. This increases the faith of the identified binding sites as functional since small molecule binders at binding sites along the structural ensemble at the LLPS-promoting regions might enable shielding of these LLPS regions from forming sticky intra/intermolecular interactions that contribute to LLPS and the following increase in transcription activity of AR-V7, linked to tumor growth. Structural characteristics of the binding sites are presented in the SI. Table S2 includes the residues that form the binding sites. Figure S6 represents the structural characteristics of the binding sites (see Figure 3).

### 3.3 Molecular Docking

Deep Dock and the Deep Docking protocol^46^ have been established to expedite the screening of molecules against a single docking site, yielding excellent results in terms of both time reduction and the enrichment of potential molecular hits. However, as previously discussed, the nature of PDPs differs from the structured regions of the proteome, often presenting multiple docking sites when targeting a PDP. This presents a challenge in the scalability of screening vast numbers of molecules; for example, in the case of the AR-V7 protein with its 41 selected docking sites, screening 1 million molecules would result in a total of 41 million screenings, a task not computationally efficient. Consequently, the careful selection of an initial pool of molecules was crucial to achieving the best possible outcomes. For this purpose, a list of 5640 compounds was randomly selected from the ChEMBL database.^36^ The binding energies of these 5640 compounds against all 41 docking sites were computed using AutoDock Vina, with more details on this process provided in the Methods section.

### 3.4 Deep Learning Multilabel Classification Model for Predicting AR-V7 Binders

The model selected for inference is based on the criteria and evaluation metrics described above. MCC values are calculated for each docking site, and the average value serves as a validation metric for selecting the optimal model. Even an MCC value of 0.5 is considered good given that the data set is highly imbalanced. This is because only the top 5% docking energies are mapped as active, giving a 95% not active and 5% active data set per docking site. For the threshold of 5% of the docking energies, the best results we obtained are presented in Table S1. Figure S2 illustrates various metrics that were monitored across different epochs during the model’s training and testing processes.

Brute force small molecule docking to screen for binders against AR-V7, comprising 41 binding sites, requires significant computational resources, which scale multiplicatively with the number of binding sites and screening compounds. Although various methods incorporate machine learning models to streamline this process,^46,47^ only a few computational methodologies are tailored to screen for binders targeting structural ensembles of PDPs.

Our method outlined in Figure 3 enabled us to rapidly screen a library of about 2 million molecules (referred to as the ‘screened set’) for which their logP and molecular weight distributions can be found in Figure S7. These compounds were randomly selected from ChEMBL in less than a minute. Out of this extensive screening, we identified molecules that were active against at least 70% of the binding sites, narrowing it down to just 402 compounds, which represents only 0.021% of the total screened molecules. These compounds were selected for redocking in AutoDock Vina against the 41 binding sites. This set is referred to as the ‘screened docking’ data set. Comprehensive results are provided in GitHub, which includes the SMILES representations of these 402 molecules, as well as their calculated binding energies for the 41 docking sites.

**Figure 4:**
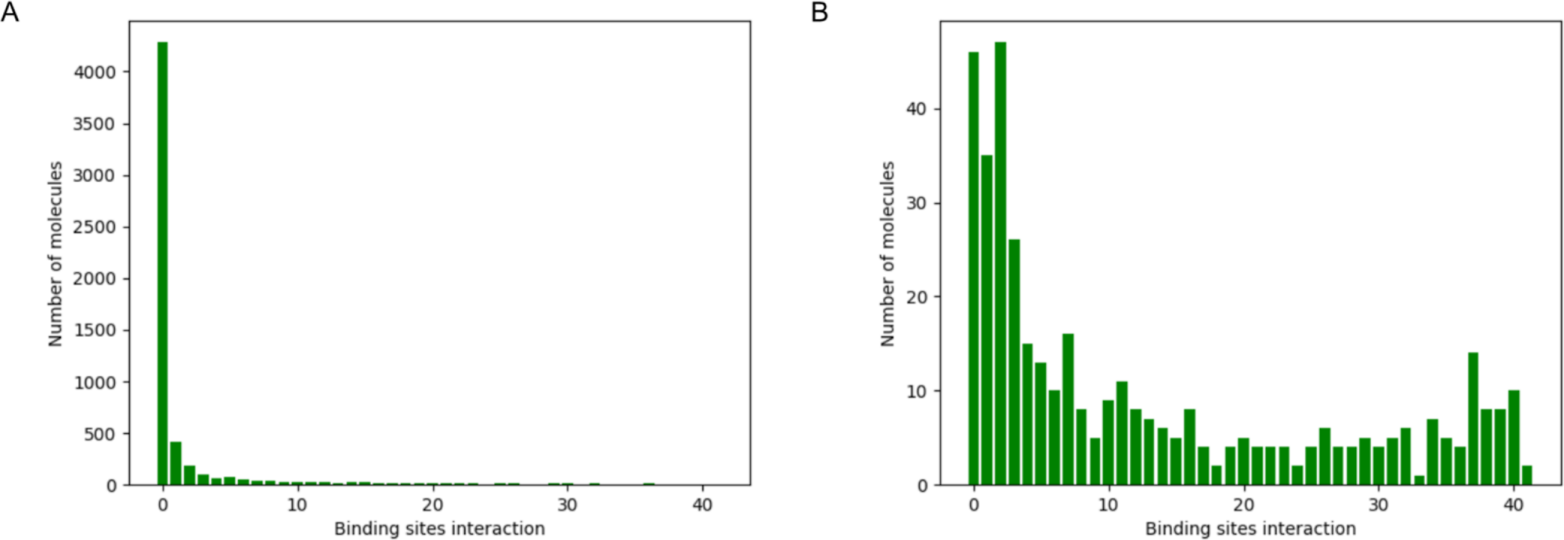
Multiple binding site hit rate. (A) Number of “active” molecules (*y*-axis) versus the number of binding sites these are active in (*x*-axis) for the naive docking data set. (B) Number of “active” molecules (*y*-axis) versus the number of binding sites these are active in (*x*-axis) for the ‘screened docking’ data set.

To further elucidate the effectiveness of our screening strategy, we carried out a comparative analysis between the 5640 molecules, which were randomly selected for training the multilabel classification model (referred to as “naive docking”) and the 402 ‘screened docking’ compounds identified through our screening and redocking. These results display the number of molecules predicted to be active against one to 41 binding sites for both sets of molecules (see Figure 4). Notably, only 57/5640 (1%) of the molecules in the naive docking set were predicted to be active against more than 30 binding sites, whereas in the ‘screened docking’ set, active molecules increased to over 70/401 (17.5%).

**Figure 5:**
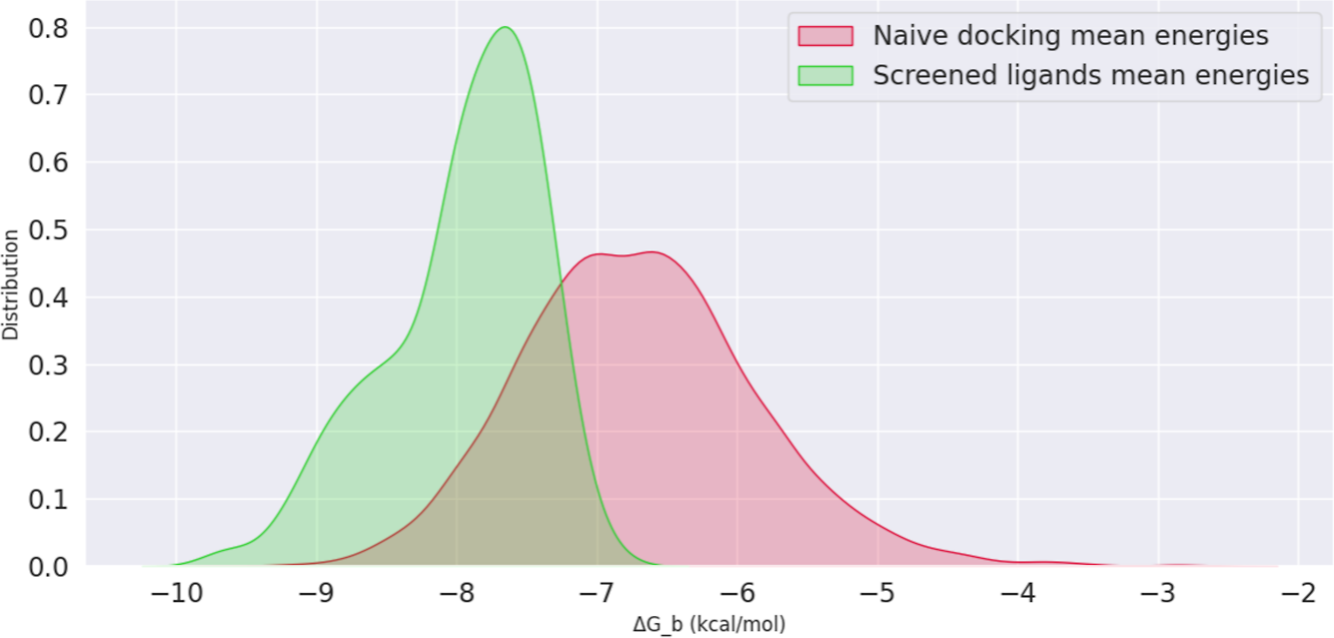
Distribution of binding energies. Kernel density estimation plot for the energies of naive docking and screened molecules.

In addition, we calculated the average binding energies across the 41 binding sites for both the naive docking and screened docking sets of molecules, respectively, and visualized these distributions by plotting their kernel density functions (see Figure 5). The molecules identified through our screening process showed significantly lower average binding energies compared to those in the naive docking set. These results indicate a greater potential for identifying effective hits for targeting the AR-V7 protein among the screened compounds, thereby highlighting the efficiency of our approach in discovering hits against PDPs like AR-V7.

To compare the QSAR model built in this section with the existing literature, we dock EPI-001 to the 41 identified AR-V7 binding sites. The EPI-001 small molecular inhibitor of the AR has been discovered by phenotypic screening, a derivative of which is in clinical trials. EPI-001 has been found to reduce transcription activity of AR-V7 when applied in μM concentrations.^32^ EPI-001 has been shown by NMR to interact with tau-5* AR-V7 regions 351–359, 397–403, and 433–437, albeit no experimental binding affinity is available. We test whether EPI-001 is active in our QSAR model and find that it is inactive in all of the 41 determined pockets found in this study, with an average binding energy of −6.3 ± 0.6 kcal/mol through the binding sites of this study. A simple explanation for this discrepancy might be that the docking energies calculated in this study by AutoDock Vina may not accurately reflect EPI-001’s true binding potential due to limitations in the docking methodology and scoring function. An alternative explanation might be a different mechanism of binding of EPI-001 to AR-V7. As shown in Figure 2C and mentioned in subsection “Binding Site Selection”, the detected binding sites in this study span mostly subregions of tau-1 (residues 150–250) and tau-5 (450–550) and only scarcely at tau-5*/tau-5 subregion 350–500, which are different than the tau-5* regions (regions 351–359, 397–403, and 433–437) that EPI-001 interacts with. In this study, we do not majorly identify binding sites in the tau-5*-EPI-001 binding AR-V7 subregions. We hypothesize that EPI-001 binds via a conformational selection mechanism with the creation of binding sites upon EPI-001 binding. However, our pipeline docks ligands in existing binding sites, akin to an induced fit mechanism. Indeed, EPI-001 has been found to induce conformational changes to AR-V7 upon binding by the formation of transient helices in regions 380–400 and 410–415.

### 3.5 Structural Ensemble Modulation of AR-V7 by ChEMBL22003

**Figure 6:**
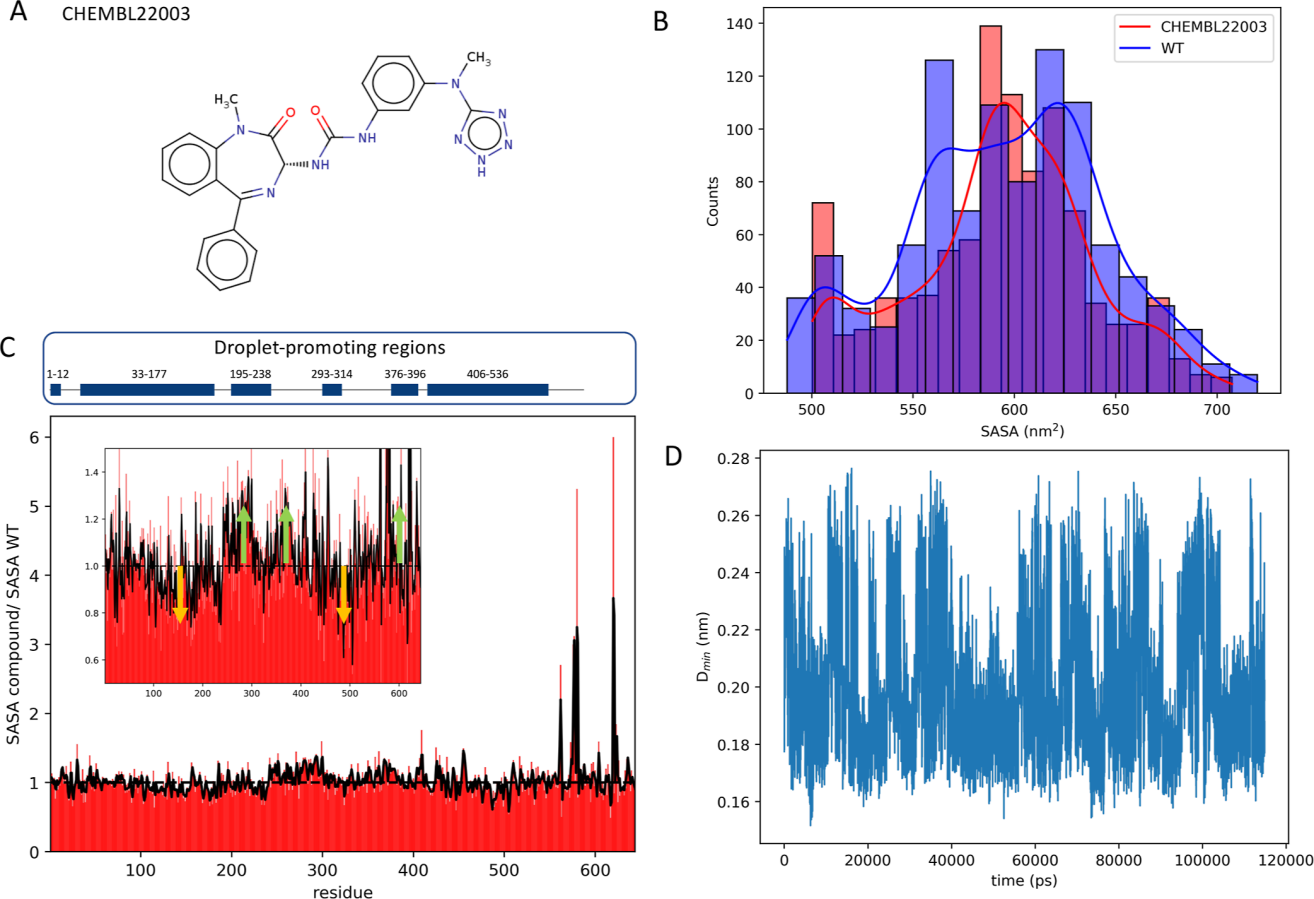
Effect of small molecule on the all-atom MD-based AR-V7 ensemble. (A) 2D structure of ChEMBL22003; (B) solvent-accessible surface area distribution of AR-V7 in the presence (red) and absence (blue) of ChEMBL22003; (C) residue-based solvent-accessible surface area of AR-V7 (green/orange arrows signify regions of increased/decreased solvent-accessible surface in the presence of the compound); and (D) AR-V7 small molecule minimum distance.

To assess the modulating effect of predicted binders targeting functional LLPS-prone regions of AR-V7, we compare the atomistic AR-V7 structural ensemble generated by PB-MetaD^26^ simulations in the presence/absence of the selected binder ChEMBL22003 (logP = 2.92, see Figure 6), found to bind to 33/41 binding sites in the redocking calculations. We find that upon binding to AR-V7, ChEMBL22003 is able to modulate AR-V7 by exhibiting slightly but significantly less absolute side-chain conformational entropy of −25.450 ± 0.005 kJ/mol·K compared to −25.370 ± 0.005 kJ/mol·K of the apo AR-V7 ensemble using the nearest neighbor approach of PDB 2ENTROPY^48^ and −24.560 ± 0.004 kJ/mol·K and −24.410 ± 0.004 kJ/mol·K when using the MIST approach of PDB 2ENTROPY.^48^ The nearest neighbor approach employed here uses default settings where no structural alignment was performed prior to the entropy calculation through the holo and apo MD structural ensembles, where no mutual information correction is included between residues. In the MIST approach, complex correlated motions are accounted for by considering only first-order mutual information for torsions closer in space than 8.0 Å and superimposing all structures on the first one. We observe a statistically significant reduction in side-chain conformational entropy upon ChEMBL22003 binding to AR-V7, with decreases of 0.08 kJ/mol·K (NN) and 0.15 kJ/mol·K (MIST). When scaled by room temperature (298 K), this corresponds to an entropic free energy contribution of 24.8 (NN) and 46.5 kJ/mol (MIST). This suggests the hypothesis that ligand binding restricts side-chain flexibility, which may contribute to the overall stabilization of the holo complex, though the overall free energy balance depends on enthalpy vs entropy compensation. The holo AR-V7 ensemble exhibits a narrower solvent-accessible surface area distribution compared to apo AR-V7 (Figure 6B) with LLPS-prone regions (1–12, 33–177, 195–238, 293–314, 376–396, and 406–536) becoming less solvent-exposed (on average 0.95625) as calculated by the average ratio between the solvent-accessible surface area of LLPS-prone regions in the holo ensemble over the ones of the apo ensemble. On the contrary, other regions (13–32, 178–194, 239–292, 315–365, 396–405, and 537–644) predicted as not LLPS-prone become more solvent-exposed (on average 1.20367) (Figure 6C), signifying a modulation of AR-V7 dynamics toward solvent shielding of LLPS-prone regions on the one hand and increased solvent exposure of other regions not related to LLPS. Particularly, regions in tau-1/tau-5 regions 100–240/470–520 become less solvent-exposed, overlapping with the tau-1/tau-5 binding sites region spanning residues 150–250/400–550 (see Figure 2C) and mostly involving LLPS-prone residues 33–177, 195–238/406–436 (see Figure 2F). On the contrary, regions of tau-1/tau-5 comprising residues 240–400/600–644 and mostly involving no LLPS-prone residues (see Figure 2F) show an increase of solvent-accessible surface area in the holo AR-V7 ensemble. Finally, we find that ChEMBL22003 remains bound to the AR-V7 binding sites, as illustrated by the small minimum distance to AR-V7 (see Figure 6D) and the small root-mean-square deviation of 0.56 ± 0.17 nm from the initial docking pose in Figure S16, further supporting the findings of tight binding by the docking calculations.

## 4 Conclusions

In this work, we illustrate a structure-based deep ensemble docking screening pipeline for functional small molecule binders that span a wide chemical space and are able to bind partially disordered proteins at multiple binding sites formed along their dynamics, which are carefully dimensionality-reduced to capture functional regions of the PDP under study. Using as a test case a full length AR-V7 transcription factor splicing variant, whose phase separation transition has been experimentally linked to the onset of tumor growth in prostate cancer, we first generate the structural ensemble of the AR-V7 monomer by using AlphaFold Metainference, identify and reduce it to 41 functional binding sites formed along its dynamics comprising low RMSF and “sticky residues” and found at LLPS-prone regions, and, subsequently, dock against these sites a medium-size data set of nearly 6000 compounds and train a multilabel neural network classifier based on binding energies of compounds per binding site that can enable to swiftly screen a larger space of 2 million ChEMBL compounds for binders at multiple binding sites. After redocking a set of 402 hit compounds from the NN screening, we find an increased multibinding site hit rate of 17.5% compared to 1% of the naive docking. To assess the modulating effect of predicted binders targeting functional LLPS-prone regions of AR-V7, we compare the atomistic AR-V7 structural ensemble generated by PB-MetaD simulations in the presence/absence of the selected binder ChEMBL22003 and show that upon binding to AR-V7, it is able to modulate AR-V7 by decreasing its conformational entropy and the width of its solvent-accessible surface area distribution compared to apo AR-V7. More importantly, LLPS-prone regions become less solvent-exposed, while other regions become more solvent-exposed. Such findings of functional modulation promote ChEMBL22003 as a potential phase separation modulator of AR-V7.

## Data Availability

The code is available on GitHub https://github.com/Pantelispanka/tar-pid. The structural ensemble of AR-V7, AF-MI preparation files, and pocket analysis is available in Zenodo DOI: 10.5281/zenodo.10985337.

## Supporting Information Available

Convergence analysis for the structural ensemble using AF-MI, validation metrics and the overall training results of the model, structure validation of five random structures from the structural ensemble, convergence of atomistic MD simulations, secondary structure prediction of atomistic MD simulations, structural properties of selected binding sites, distribution of logP and molecular weight of naive docking and screened docking small molecule data sets, binding energy distributions of naive docking and screened small molecule data sets, pose stability, and residues involved in the 41 selected binding sites (PDF)
