# Supplementary material for: Small Molecules Targeting the Structural Dynamics of AR-V7 Partially Disordered Proteins Using Deep Ensemble Docking
Source: J Chem Theory Comput. 2025 Apr 15;21(9):4898–909. doi: 10.1021/acs.jctc.5c00171 (PMC12080126; doi:10.1021/acs.jctc.5c00171)
Supplement: Supplementary file 1 — ct5c00171_si_001.pdf [file ct5c00171_si_001.pdf]

Supporting Information

Small molecules targeting the structural dynamics of AR-V7 partially disordered protein using deep ensemble docking

Pantelis Karatzas[1],*,† Z. Faidon Brotzakis[1],*,‡,¶ and Haralambos Sarimveis*,†

†*School of Chemical Engineering, National Technical University of Athens, 9 Heroon Polytechniou Street, 15780 Athens, Greece*

‡*Institute of Bioinnovation (IBI), Biomedical Science Research Center Alexander Fleming, 34 Fleming Street, 16672, Vari, Greece*

¶*Centre for Misfolding Diseases, Department of Chemistry, University of Cambridge, Lensfield Road, Cambridge CB2 1EW, UK*

E-mail: pantelispanka@gmail.com; brotzakis@fleming.gr; hsarimv@central.ntua.gr

[1]Equal contribution

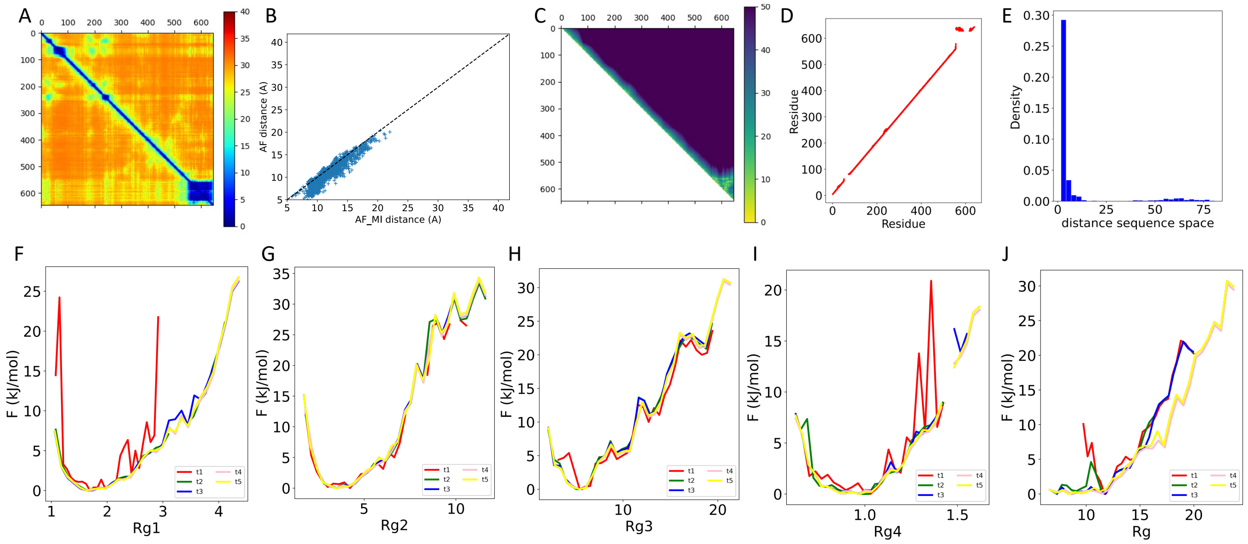

Figure S1: AlphaFold Metainference statistics. A) Residue based predicted alignment error in Angstong. B) AlphaFold predicted and AF-MI restrained distances. C) residue pairwise distance map of the AR-V7 structural ensemble. D) Inter-residue distances used as restraints in AF-MI, E) Probability distribution of sequence space distance of the restraint distances F,G,H,I) Time dependent free energies of radii of gyration of the disordered segments of AR-V7 (Rg1, Rg2, Rg3, Rg4) and J) of the radius of gyration of the entire AR-V7.

Table S1: Forward neural network model validation metrics.

Cavities	204_KAM, 134_KAC, 22_KAJ, 80_KAD, 154_KAN, 296_KAW, 92_KAM, 140_KAC, 260_KAF, 192_KAO, 220_KAH, 310_KAQ, 104_KAF, 268_KAL, 304_KAQ, 342_KAI, 290_KBA, 322_KAK, 210_KAF, 34_KAS, 178_KAA, 56_KAS, 58_KAF, 72_KAI, 8_KAI, 200_KAR, 134_KAH, 212_KAL, 286_KAM, 182_KAD, 216_KAN, 284_KAJ, 148_KAG, 340_KAH, 314_KAF, 222_KAV, 140_KAD, 168_KAK, 22_KAI, 156_KAE, 216_KAA
Zero One Loss	0.58
Hamming Loss	0.10
Accuracy	0.896, 0.888, 0.901, 0.889, 0.883, 0.897, 0.874, 0.879, 0.911, 0.893, 0.865, 0.882, 0.881, 0.901, 0.900, 0.888, 0.915, 0.903, 0.894, 0.870, 0.876, 0.888, 0.877, 0.909, 0.888, 0.898, 0.901, 0.898, 0.917, 0.894, 0.880, 0.893, 0.898, 0.878, 0.914, 0.882, 0.893, 0.903, 0.890, 0.875, 0.900
MCC	0.437, 0.602, 0.643, 0.467, 0.548, 0.621, 0.486, 0.527, 0.657, 0.591, 0.468, 0.463, 0.496, 0.624, 0.620, 0.440, 0.624, 0.676, 0.556, 0.532, 0.411, 0.533, 0.560, 0.685, 0.548, 0.0, 0.651, 0.504, 0.682, 0.561, 0.545, 0.646, 0.600, 0.555, 0.664, 0.535, 0.478, 0.652, 0.534, 0.309, 0.615
Epoch loss	0.23

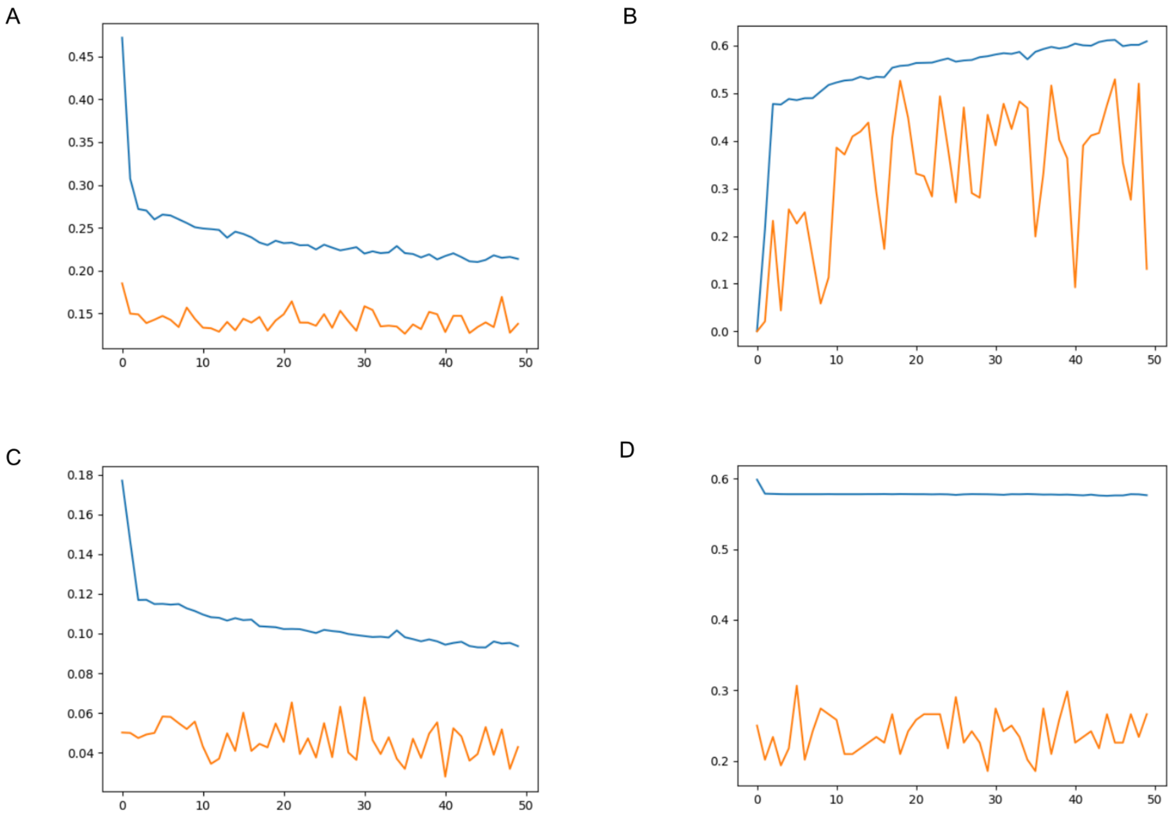

Figure S2: Training the model. A) Binary cross entropy loss per epoch for train and test, B) Average MCC per epoch, C) Hamming loss per epoch, D) Zero one loss per epoch for the top 5% of the docking energies. Blue line corresponds to train set and red to test set.

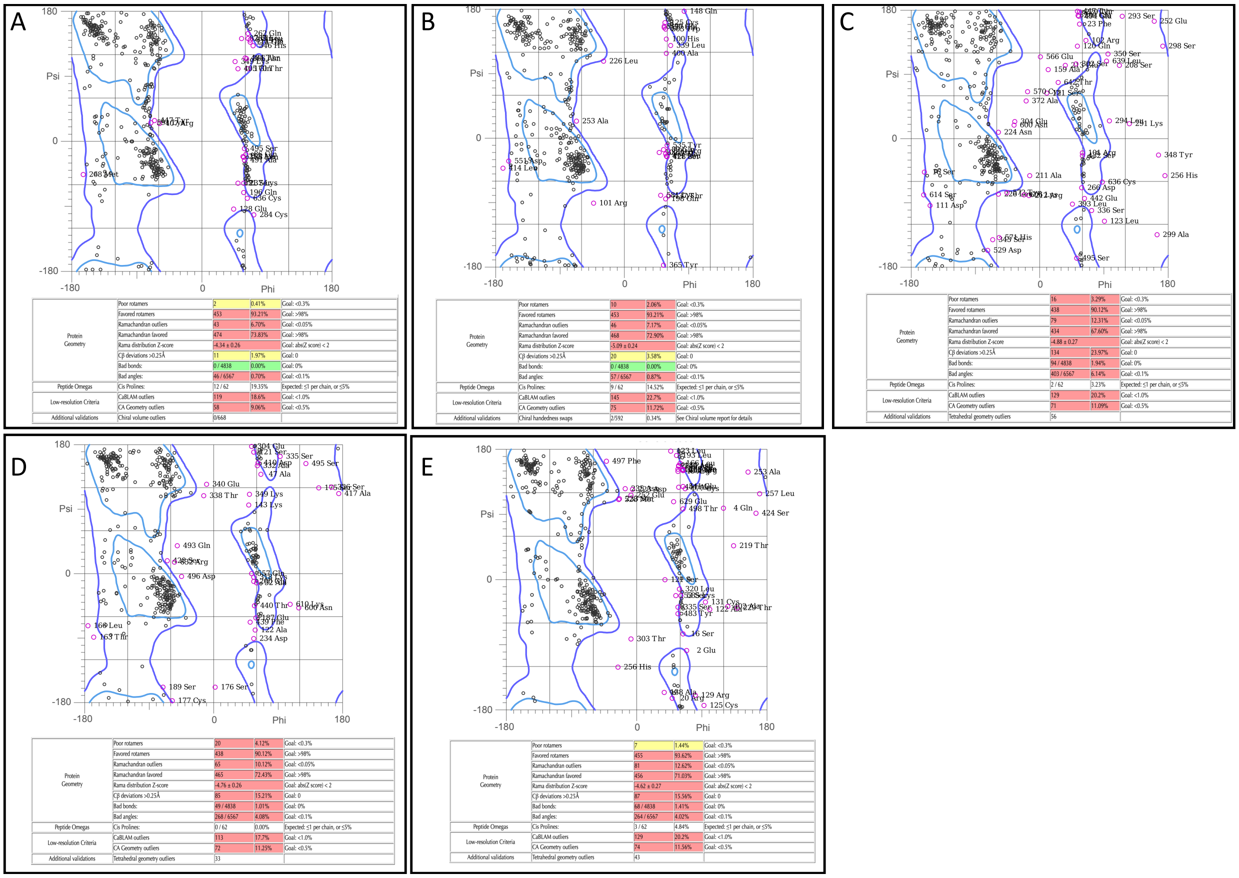

Figure S3: Structure validation of five random structures from the structural ensemble. A-E) Ramachandran plots and structure validation metrics for random structures from the structural ensemble.

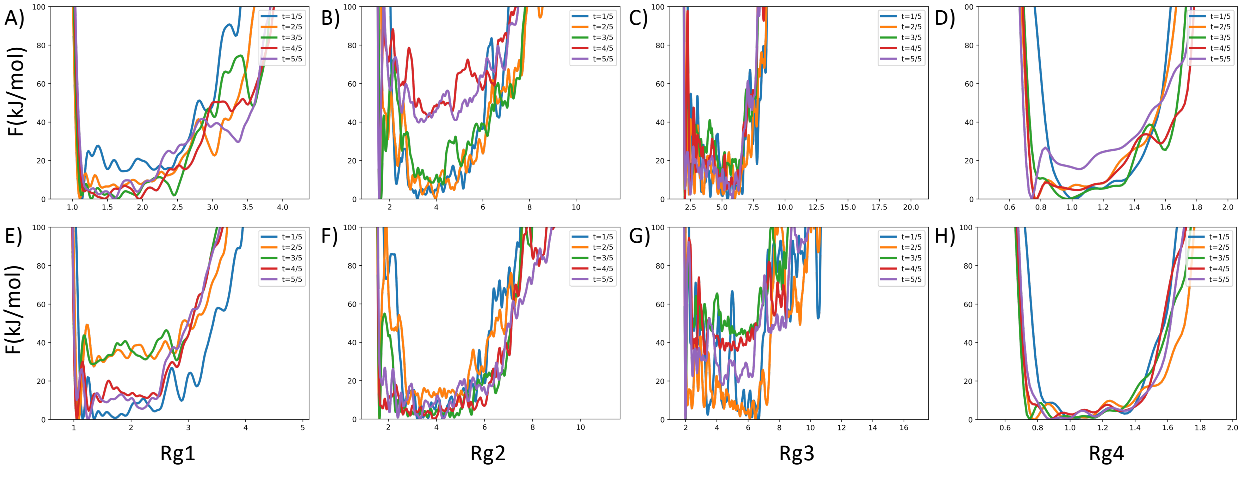

Figure S4: Convergence of atomistic MD along four collective variables Rg1,Rg2,Rg3,Rg4 used for the holo AR-V7 (A-D) and the apo AR-V7 (E-H).

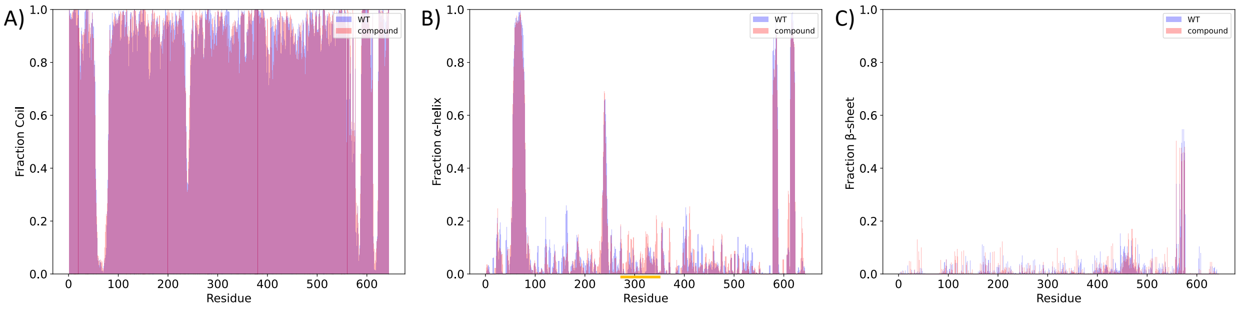

Figure S5: Residue based A) coil, B)α-helix, C) β-sheet secondary structure prediction by atomistic MD for the apo AR-V7 in blue and holo AR-V7 in red.

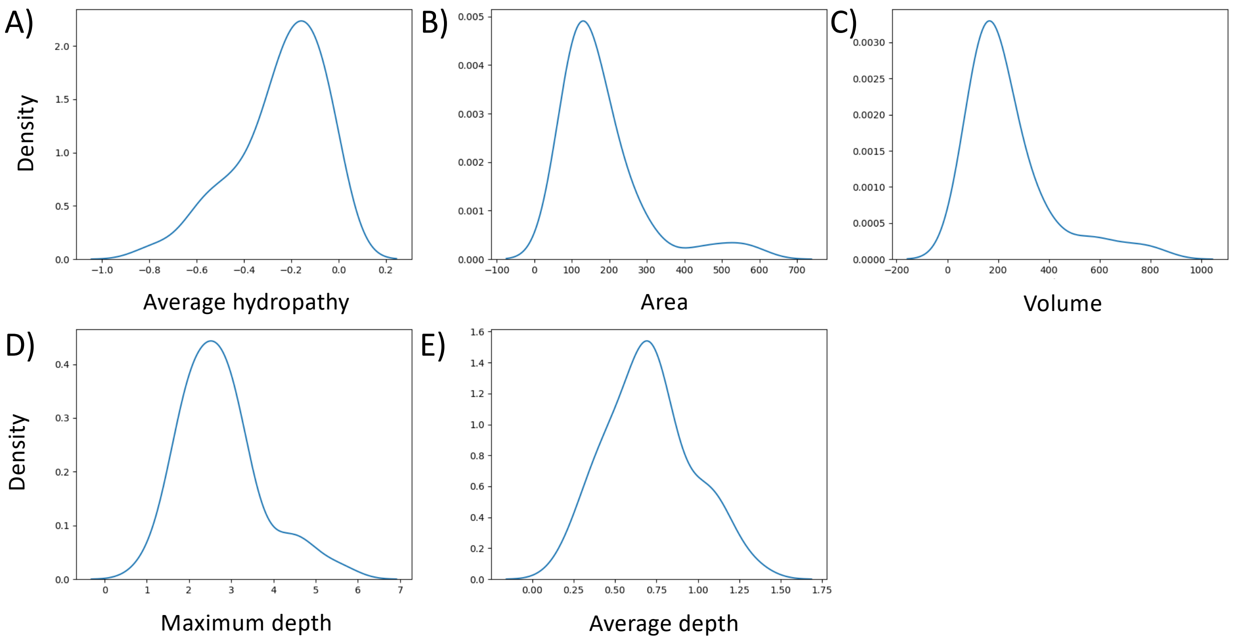

Figure S6: Structural properties of the detected binding sites. A) Average hydropathy, B) Area, C) Volume, D) Maximum depth, E) Average depth.

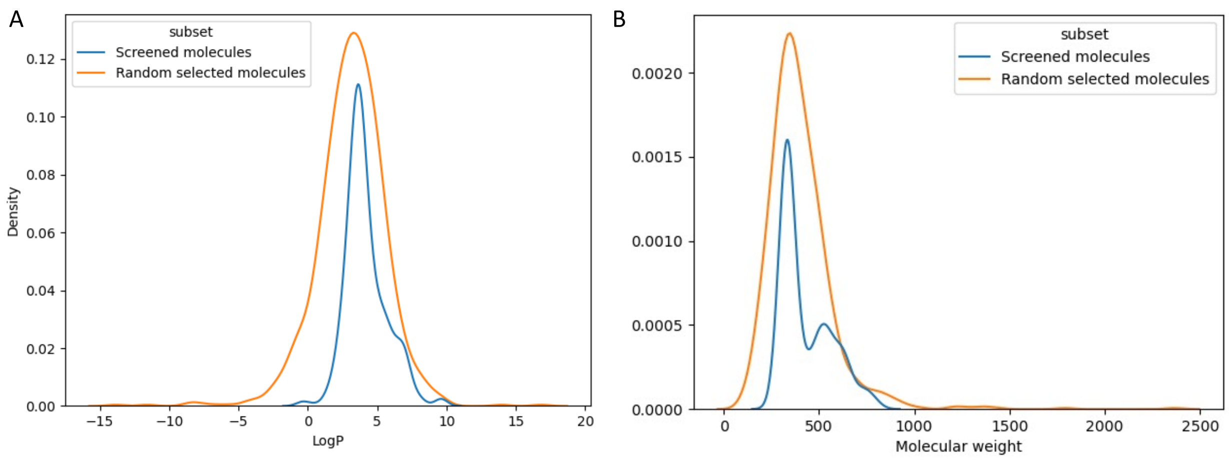

Figure S7: Distribution of A) LogP and B) MW of the screened and 1000 randomly selected molecules from CHEMBL.

204_KAM	[['209', 'A', 'GLY'], ['210', 'A', 'ARG'], ['211', 'A', 'ALA'], ['213', 'A', 'GLU'], ['214', 'A', 'ALA'], ['215', 'A', 'SER'], ['216', 'A', 'GLY'], ['217', 'A', 'ALA'], ['226', 'A', 'LEU'], ['227', 'A', 'GLY'], ['228', 'A', 'GLY'], ['229', 'A', 'THR'], ['231', 'A', 'THR'], ['232', 'A', 'ILE']]
134_KAC	[['174', 'A', 'LEU'], ['175', 'A', 'SER'], ['176', 'A', 'SER'], ['177', 'A', 'CYS'], ['178', 'A', 'SER'], ['179', 'A', 'ALA'], ['181', 'A', 'LEU'], ['190', 'A', 'THR'], ['191', 'A', 'MET'], ['192', 'A', 'GLN'], ['193', 'A', 'LEU'], ['194', 'A', 'LEU'], ['195', 'A', 'GLN'], ['196', 'A', 'GLN'], ['197', 'A', 'GLN'], ['198', 'A', 'GLN'], ['199', 'A', 'GLN'], ['200', 'A', 'GLU'], ['206', 'A', 'SER']]

22_KAJ	[['490', 'A', 'LEU'], ['491', 'A', 'ALA'], ['492', 'A', 'GLY'], ['493', 'A', 'GLN'], ['494', 'A', 'GLU'], ['495', 'A', 'SER'], ['496', 'A', 'ASP'], ['497', 'A', 'PHE'], ['498', 'A', 'THR'], ['499', 'A', 'ALA'], ['500', 'A', 'PRO'], ['502', 'A', 'VAL'], ['508', 'A', 'MET'], ['509', 'A', 'VAL'], ['510', 'A', 'SER'], ['511', 'A', 'ARG']]
80_KAD	[['160', 'A', 'ALA'], ['161', 'A', 'PRO'], ['162', 'A', 'SER'], ['163', 'A', 'THR'], ['164', 'A', 'LEU'], ['165', 'A', 'SER'], ['166', 'A', 'LEU'], ['167', 'A', 'LEU'], ['168', 'A', 'GLY'], ['177', 'A', 'CYS'], ['178', 'A', 'SER'], ['179', 'A', 'ALA'], ['180', 'A', 'ASP'], ['181', 'A', 'LEU'], ['182', 'A', 'LYS'], ['183', 'A', 'ASP'], ['184', 'A', 'ILE'], ['185', 'A', 'LEU']]
154_KAN	[['180', 'A', 'ASP'], ['181', 'A', 'LEU'], ['182', 'A', 'LYS'], ['183', 'A', 'ASP'], ['185', 'A', 'LEU'], ['186', 'A', 'SER'], ['187', 'A', 'GLU'], ['188', 'A', 'ALA'], ['189', 'A', 'SER'], ['190', 'A', 'THR'], ['191', 'A', 'MET'], ['192', 'A', 'GLN'], ['193', 'A', 'LEU'], ['197', 'A', 'GLN'], ['198', 'A', 'GLN'], ['199', 'A', 'GLN'], ['200', 'A', 'GLU'], ['201', 'A', 'ALA'], ['203', 'A', 'SER'], ['204', 'A', 'GLU'], ['206', 'A', 'SER']]

296_KAW	[['497', 'A', 'PHE'], ['498', 'A', 'THR'], ['499', 'A', 'ALA'], ['511', 'A', 'ARG'], ['512', 'A', 'VAL'], ['513', 'A', 'PRO'], ['517', 'A', 'PRO'], ['518', 'A', 'THR'], ['519', 'A', 'CYS'], ['520', 'A', 'VAL'], ['521', 'A', 'LYS'], ['522', 'A', 'SER'], ['523', 'A', 'GLU'], ['524', 'A', 'MET'], ['525', 'A', 'GLY'], ['526', 'A', 'PRO'], ['527', 'A', 'TRP'], ['528', 'A', 'MET'], ['542', 'A', 'THR'], ['543', 'A', 'ALA'], ['544', 'A', 'ARG'], ['545', 'A', 'ASP'], ['546', 'A', 'HIS'], ['547', 'A', 'VAL'], ['548', 'A', 'LEU'], ['550', 'A', 'ILE'], ['552', 'A', 'TYR'], ['553', 'A', 'TYR'], ['556', 'A', 'PRO'], ['557', 'A', 'GLN'], ['570', 'A', 'CYS'], ['573', 'A', 'GLY'], ['574', 'A', 'ALA'], ['575', 'A', 'LEU'], ['623', 'A', 'ALA'], ['624', 'A', 'GLY'], ['630', 'A', 'LYS']]
92_KAM	[['179', 'A', 'ALA'], ['180', 'A', 'ASP'], ['181', 'A', 'LEU'], ['185', 'A', 'LEU'], ['186', 'A', 'SER'], ['188', 'A', 'ALA'], ['192', 'A', 'GLN'], ['193', 'A', 'LEU'], ['194', 'A', 'LEU'], ['195', 'A', 'GLN'], ['196', 'A', 'GLN'], ['197', 'A', 'GLN'], ['198', 'A', 'GLN'], ['202', 'A', 'VAL']]

140_KAC	[['139', 'A', 'VAL'], ['140', 'A', 'ALA'], ['159', 'A', 'ALA'], ['160', 'A', 'ALA'], ['161', 'A', 'PRO'], ['162', 'A', 'SER'], ['163', 'A', 'THR'], ['164', 'A', 'LEU'], ['166', 'A', 'LEU'], ['185', 'A', 'LEU'], ['186', 'A', 'SER'], ['187', 'A', 'GLU'], ['188', 'A', 'ALA'], ['189', 'A', 'SER'], ['190', 'A', 'THR'], ['191', 'A', 'MET'], ['192', 'A', 'GLN'], ['193', 'A', 'LEU']]
260_KAF	[['474', 'A', 'GLU'], ['475', 'A', 'ALA'], ['477', 'A', 'ALA'], ['478', 'A', 'VAL'], ['479', 'A', 'ALA'], ['480', 'A', 'PRO'], ['481', 'A', 'TYR'], ['482', 'A', 'GLY'], ['483', 'A', 'TYR'], ['484', 'A', 'THR'], ['485', 'A', 'ARG'], ['486', 'A', 'PRO'], ['487', 'A', 'PRO'], ['488', 'A', 'GLN'], ['489', 'A', 'GLY'], ['490', 'A', 'LEU'], ['491', 'A', 'ALA'], ['497', 'A', 'PHE'], ['500', 'A', 'PRO'], ['501', 'A', 'ASP'], ['502', 'A', 'VAL'], ['511', 'A', 'ARG'], ['516', 'A', 'SER'], ['517', 'A', 'PRO'], ['518', 'A', 'THR'], ['521', 'A', 'LYS'], ['526', 'A', 'PRO']]
192_KAO	[['476', 'A', 'GLY'], ['477', 'A', 'ALA'], ['479', 'A', 'ALA'], ['480', 'A', 'PRO'], ['481', 'A', 'TYR'], ['488', 'A', 'GLN'], ['493', 'A', 'GLN'], ['494', 'A', 'GLU'], ['495', 'A', 'SER'], ['496', 'A', 'ASP'], ['497', 'A', 'PHE'], ['498', 'A', 'THR'], ['501', 'A', 'ASP'], ['502', 'A', 'VAL'], ['503', 'A', 'TRP'], ['504', 'A', 'TYR']]

220_KAH	[['489', 'A', 'GLY'], ['490', 'A', 'LEU'], ['491', 'A', 'ALA'], ['496', 'A', 'ASP'], ['497', 'A', 'PHE'], ['498', 'A', 'THR'], ['499', 'A', 'ALA'], ['500', 'A', 'PRO'], ['506', 'A', 'GLY'], ['507', 'A', 'GLY'], ['508', 'A', 'MET'], ['509', 'A', 'VAL'], ['511', 'A', 'ARG']]
310_KAQ	[['479', 'A', 'ALA'], ['481', 'A', 'TYR'], ['482', 'A', 'GLY'], ['483', 'A', 'TYR'], ['484', 'A', 'THR'], ['486', 'A', 'PRO'], ['487', 'A', 'PRO'], ['488', 'A', 'GLN'], ['489', 'A', 'GLY'], ['491', 'A', 'ALA'], ['492', 'A', 'GLY'], ['493', 'A', 'GLN'], ['494', 'A', 'GLU'], ['495', 'A', 'SER'], ['496', 'A', 'ASP'], ['497', 'A', 'PHE'], ['498', 'A', 'THR'], ['499', 'A', 'ALA'], ['500', 'A', 'PRO'], ['502', 'A', 'VAL']]
104_KAF	[['444', 'A', 'GLY'], ['445', 'A', 'GLN'], ['446', 'A', 'LEU'], ['447', 'A', 'TYR'], ['449', 'A', 'PRO'], ['486', 'A', 'PRO'], ['487', 'A', 'PRO'], ['488', 'A', 'GLN'], ['490', 'A', 'LEU'], ['491', 'A', 'ALA'], ['492', 'A', 'GLY'], ['493', 'A', 'GLN'], ['494', 'A', 'GLU'], ['496', 'A', 'ASP'], ['497', 'A', 'PHE'], ['498', 'A', 'THR'], ['499', 'A', 'ALA'], ['500', 'A', 'PRO'], ['501', 'A', 'ASP'], ['502', 'A', 'VAL'], ['503', 'A', 'TRP'], ['504', 'A', 'TYR'], ['505', 'A', 'PRO'], ['506', 'A', 'GLY']]

268_KAL	[['476', 'A', 'GLY'], ['477', 'A', 'ALA'], ['479', 'A', 'ALA'], ['480', 'A', 'PRO'], ['481', 'A', 'TYR'], ['482', 'A', 'GLY'], ['483', 'A', 'TYR'], ['484', 'A', 'THR'], ['485', 'A', 'ARG'], ['486', 'A', 'PRO'], ['487', 'A', 'PRO'], ['491', 'A', 'ALA'], ['492', 'A', 'GLY'], ['495', 'A', 'SER'], ['496', 'A', 'ASP'], ['497', 'A', 'PHE']]
304_KAQ	[['170', 'A', 'THR'], ['171', 'A', 'PHE'], ['173', 'A', 'GLY'], ['174', 'A', 'LEU'], ['175', 'A', 'SER'], ['177', 'A', 'CYS'], ['179', 'A', 'ALA'], ['181', 'A', 'LEU'], ['182', 'A', 'LYS'], ['183', 'A', 'ASP'], ['185', 'A', 'LEU'], ['186', 'A', 'SER'], ['187', 'A', 'GLU'], ['188', 'A', 'ALA'], ['189', 'A', 'SER'], ['190', 'A', 'THR'], ['191', 'A', 'MET'], ['192', 'A', 'GLN'], ['193', 'A', 'LEU'], ['194', 'A', 'LEU'], ['195', 'A', 'GLN'], ['196', 'A', 'GLN'], ['197', 'A', 'GLN'], ['198', 'A', 'GLN'], ['200', 'A', 'GLU']]
342_KAI	[['185', 'A', 'LEU'], ['189', 'A', 'SER'], ['190', 'A', 'THR'], ['191', 'A', 'MET'], ['192', 'A', 'GLN'], ['193', 'A', 'LEU'], ['194', 'A', 'LEU'], ['197', 'A', 'GLN'], ['198', 'A', 'GLN'], ['199', 'A', 'GLN'], ['200', 'A', 'GLU'], ['201', 'A', 'ALA'], ['202', 'A', 'VAL'], ['203', 'A', 'SER'], ['204', 'A', 'GLU']]

290_KBA	[['364', 'A', 'TYR'], ['487', 'A', 'PRO'], ['488', 'A', 'GLN'], ['489', 'A', 'GLY'], ['490', 'A', 'LEU'], ['491', 'A', 'ALA'], ['492', 'A', 'GLY'], ['493', 'A', 'GLN'], ['494', 'A', 'GLU'], ['495', 'A', 'SER'], ['496', 'A', 'ASP'], ['497', 'A', 'PHE'], ['504', 'A', 'TYR'], ['511', 'A', 'ARG']]
322_KAK	[['487', 'A', 'PRO'], ['488', 'A', 'GLN'], ['507', 'A', 'GLY'], ['508', 'A', 'MET'], ['509', 'A', 'VAL'], ['510', 'A', 'SER'], ['511', 'A', 'ARG'], ['512', 'A', 'VAL'], ['513', 'A', 'PRO'], ['514', 'A', 'TYR'], ['515', 'A', 'PRO'], ['516', 'A', 'SER'], ['518', 'A', 'THR'], ['519', 'A', 'CYS'], ['520', 'A', 'VAL'], ['521', 'A', 'LYS']]
210_KAF	[['483', 'A', 'TYR'], ['485', 'A', 'ARG'], ['486', 'A', 'PRO'], ['487', 'A', 'PRO'], ['488', 'A', 'GLN'], ['491', 'A', 'ALA'], ['492', 'A', 'GLY'], ['493', 'A', 'GLN'], ['494', 'A', 'GLU'], ['495', 'A', 'SER'], ['497', 'A', 'PHE'], ['498', 'A', 'THR'], ['499', 'A', 'ALA'], ['500', 'A', 'PRO'], ['501', 'A', 'ASP'], ['502', 'A', 'VAL'], ['503', 'A', 'TRP'], ['512', 'A', 'VAL'], ['513', 'A', 'PRO'], ['514', 'A', 'TYR']]
34_KAS	[['158', 'A', 'SER'], ['161', 'A', 'PRO'], ['165', 'A', 'SER'], ['166', 'A', 'LEU'], ['172', 'A', 'PRO'], ['180', 'A', 'ASP'], ['184', 'A', 'ILE'], ['185', 'A', 'LEU'], ['190', 'A', 'THR'], ['191', 'A', 'MET'], ['198', 'A', 'GLN'], ['201', 'A', 'ALA'], ['202', 'A', 'VAL'], ['203', 'A', 'SER'], ['204', 'A', 'GLU'], ['205', 'A', 'GLY'], ['206', 'A', 'SER']]

178_KAA	[['478', 'A', 'VAL'], ['479', 'A', 'ALA'], ['480', 'A', 'PRO'], ['481', 'A', 'TYR'], ['482', 'A', 'GLY'], ['483', 'A', 'TYR'], ['484', 'A', 'THR'], ['499', 'A', 'ALA'], ['500', 'A', 'PRO'], ['504', 'A', 'TYR'], ['508', 'A', 'MET'], ['509', 'A', 'VAL'], ['510', 'A', 'SER'], ['511', 'A', 'ARG'], ['512', 'A', 'VAL']]
56_KAS	[['200', 'A', 'GLU'], ['201', 'A', 'ALA'], ['202', 'A', 'VAL'], ['207', 'A', 'SER'], ['208', 'A', 'SER'], ['209', 'A', 'GLY'], ['210', 'A', 'ARG'], ['211', 'A', 'ALA'], ['212', 'A', 'ARG'], ['213', 'A', 'GLU'], ['214', 'A', 'ALA'], ['215', 'A', 'SER'], ['222', 'A', 'LYS'], ['223', 'A', 'ASP'], ['224', 'A', 'ASN'], ['225', 'A', 'TYR'], ['226', 'A', 'LEU'], ['229', 'A', 'THR']]
58_KAF	[['470', 'A', 'GLY'], ['471', 'A', 'GLY'], ['472', 'A', 'GLY'], ['473', 'A', 'GLY'], ['474', 'A', 'GLU'], ['475', 'A', 'ALA'], ['476', 'A', 'GLY'], ['477', 'A', 'ALA'], ['478', 'A', 'VAL'], ['479', 'A', 'ALA'], ['480', 'A', 'PRO'], ['481', 'A', 'TYR'], ['482', 'A', 'GLY'], ['483', 'A', 'TYR'], ['484', 'A', 'THR'], ['485', 'A', 'ARG'], ['486', 'A', 'PRO'], ['487', 'A', 'PRO'], ['490', 'A', 'LEU'], ['491', 'A', 'ALA'], ['492', 'A', 'GLY'], ['495', 'A', 'SER'], ['496', 'A', 'ASP'], ['497', 'A', 'PHE'], ['498', 'A', 'THR'], ['499', 'A', 'ALA'], ['500', 'A', 'PRO']]

72_KAI	[['171', 'A', 'PHE'], ['172', 'A', 'PRO'], ['173', 'A', 'GLY'], ['174', 'A', 'LEU'], ['178', 'A', 'SER'], ['179', 'A', 'ALA'], ['180', 'A', 'ASP'], ['184', 'A', 'ILE'], ['185', 'A', 'LEU'], ['189', 'A', 'SER'], ['190', 'A', 'THR'], ['191', 'A', 'MET'], ['222', 'A', 'LYS'], ['223', 'A', 'ASP'], ['224', 'A', 'ASN'], ['225', 'A', 'TYR'], ['227', 'A', 'GLY'], ['228', 'A', 'GLY'], ['234', 'A', 'ASP'], ['235', 'A', 'ASN']]
8_KAI	[['424', 'A', 'SER'], ['425', 'A', 'GLY'], ['426', 'A', 'SER'], ['438', 'A', 'LEU'], ['439', 'A', 'PHE'], ['440', 'A', 'THR'], ['493', 'A', 'GLN'], ['494', 'A', 'GLU'], ['495', 'A', 'SER'], ['496', 'A', 'ASP'], ['497', 'A', 'PHE'], ['498', 'A', 'THR'], ['499', 'A', 'ALA'], ['500', 'A', 'PRO'], ['501', 'A', 'ASP'], ['502', 'A', 'VAL'], ['503', 'A', 'TRP'], ['504', 'A', 'TYR'], ['505', 'A', 'PRO'], ['508', 'A', 'MET'], ['509', 'A', 'VAL'], ['510', 'A', 'SER'], ['511', 'A', 'ARG']]
200_KAR	[['173', 'A', 'GLY'], ['174', 'A', 'LEU'], ['175', 'A', 'SER'], ['176', 'A', 'SER'], ['177', 'A', 'CYS'], ['178', 'A', 'SER'], ['179', 'A', 'ALA'], ['180', 'A', 'ASP'], ['185', 'A', 'LEU'], ['186', 'A', 'SER'], ['187', 'A', 'GLU'], ['188', 'A', 'ALA'], ['189', 'A', 'SER'], ['193', 'A', 'LEU'], ['194', 'A', 'LEU'], ['195', 'A', 'GLN']]

134_KAH	[['394', 'A', 'ASP'], ['395', 'A', 'TYR'], ['396', 'A', 'GLY'], ['398', 'A', 'ALA'], ['438', 'A', 'LEU'], ['439', 'A', 'PHE'], ['440', 'A', 'THR'], ['441', 'A', 'ALA'], ['447', 'A', 'TYR'], ['448', 'A', 'GLY'], ['449', 'A', 'PRO'], ['460', 'A', 'GLY'], ['461', 'A', 'GLY'], ['462', 'A', 'GLY'], ['470', 'A', 'GLY'], ['471', 'A', 'GLY'], ['472', 'A', 'GLY'], ['473', 'A', 'GLY'], ['475', 'A', 'ALA'], ['476', 'A', 'GLY'], ['477', 'A', 'ALA'], ['478', 'A', 'VAL'], ['479', 'A', 'ALA'], ['482', 'A', 'GLY'], ['483', 'A', 'TYR'], ['485', 'A', 'ARG'], ['486', 'A', 'PRO'], ['487', 'A', 'PRO'], ['488', 'A', 'GLN'], ['489', 'A', 'GLY']]
212_KAL	[['450', 'A', 'CYS'], ['452', 'A', 'GLY'], ['453', 'A', 'GLY'], ['455', 'A', 'GLY'], ['461', 'A', 'GLY'], ['462', 'A', 'GLY'], ['463', 'A', 'GLY'], ['465', 'A', 'GLY'], ['466', 'A', 'GLY'], ['480', 'A', 'PRO'], ['481', 'A', 'TYR'], ['482', 'A', 'GLY'], ['483', 'A', 'TYR'], ['484', 'A', 'THR'], ['485', 'A', 'ARG'], ['494', 'A', 'GLU'], ['495', 'A', 'SER'], ['496', 'A', 'ASP'], ['497', 'A', 'PHE'], ['498', 'A', 'THR']]
286_KAM	[['468', 'A', 'GLY'], ['469', 'A', 'GLY'], ['470', 'A', 'GLY'], ['473', 'A', 'GLY'], ['474', 'A', 'GLU'], ['475', 'A', 'ALA'], ['476', 'A', 'GLY'], ['477', 'A', 'ALA'], ['478', 'A', 'VAL'], ['483', 'A', 'TYR'], ['493', 'A', 'GLN'], ['502', 'A', 'VAL'], ['506', 'A', 'GLY'], ['507', 'A', 'GLY'], ['508', 'A', 'MET']]

182_KAD	[['164', 'A', 'LEU'], ['165', 'A', 'SER'], ['166', 'A', 'LEU'], ['167', 'A', 'LEU'], ['175', 'A', 'SER'], ['179', 'A', 'ALA'], ['180', 'A', 'ASP'], ['181', 'A', 'LEU'], ['182', 'A', 'LYS'], ['183', 'A', 'ASP'], ['184', 'A', 'ILE'], ['185', 'A', 'LEU'], ['186', 'A', 'SER'], ['188', 'A', 'ALA'], ['191', 'A', 'MET'], ['192', 'A', 'GLN'], ['193', 'A', 'LEU'], ['194', 'A', 'LEU'], ['195', 'A', 'GLN'], ['196', 'A', 'GLN'], ['197', 'A', 'GLN'], ['198', 'A', 'GLN'], ['199', 'A', 'GLN'], ['200', 'A', 'GLU'], ['201', 'A', 'ALA'], ['203', 'A', 'SER'], ['204', 'A', 'GLU'], ['207', 'A', 'SER'], ['238', 'A', 'GLU'], ['241', 'A', 'LYS'], ['242', 'A', 'ALA'], ['244', 'A', 'SER'], ['245', 'A', 'VAL'], ['250', 'A', 'GLY'], ['251', 'A', 'VAL'], ['252', 'A', 'GLU'], ['253', 'A', 'ALA'], ['255', 'A', 'GLU'], ['256', 'A', 'HIS'], ['257', 'A', 'LEU'], ['258', 'A', 'SER'], ['259', 'A', 'PRO'], ['260', 'A', 'GLY'], ['261', 'A', 'GLU'], ['262', 'A', 'GLN'], ['263', 'A', 'LEU'], ['264', 'A', 'ARG']]
216_KAN	[['149', 'A', 'LEU'], ['150', 'A', 'PRO'], ['151', 'A', 'ALA'], ['152', 'A', 'PRO'], ['153', 'A', 'PRO'], ['155', 'A', 'GLU'], ['156', 'A', 'ASP'], ['171', 'A', 'PHE'], ['172', 'A', 'PRO'], ['173', 'A', 'GLY'], ['183', 'A', 'ASP'], ['187', 'A', 'GLU'], ['188', 'A', 'ALA'], ['189', 'A', 'SER'], ['190', 'A', 'THR'], ['192', 'A', 'GLN'], ['193', 'A', 'LEU'], ['195', 'A', 'GLN'], ['196', 'A', 'GLN'], ['197', 'A', 'GLN'], ['198', 'A', 'GLN'], ['199', 'A', 'GLN'], ['204', 'A', 'GLU']]

284_KAJ	[['426', 'A', 'SER'], ['427', 'A', 'PRO'], ['428', 'A', 'SER'], ['429', 'A', 'ALA'], ['431', 'A', 'ALA'], ['433', 'A', 'SER'], ['434', 'A', 'SER'], ['435', 'A', 'TRP'], ['436', 'A', 'HIS'], ['459', 'A', 'GLY'], ['460', 'A', 'GLY'], ['466', 'A', 'GLY'], ['468', 'A', 'GLY'], ['469', 'A', 'GLY'], ['470', 'A', 'GLY'], ['471', 'A', 'GLY'], ['472', 'A', 'GLY'], ['473', 'A', 'GLY'], ['475', 'A', 'ALA'], ['476', 'A', 'GLY'], ['483', 'A', 'TYR'], ['484', 'A', 'THR'], ['485', 'A', 'ARG']]
148_KAG	[['495', 'A', 'SER'], ['496', 'A', 'ASP'], ['497', 'A', 'PHE'], ['498', 'A', 'THR'], ['499', 'A', 'ALA'], ['500', 'A', 'PRO'], ['501', 'A', 'ASP'], ['503', 'A', 'TRP'], ['504', 'A', 'TYR'], ['505', 'A', 'PRO'], ['510', 'A', 'SER'], ['511', 'A', 'ARG'], ['512', 'A', 'VAL'], ['513', 'A', 'PRO'], ['514', 'A', 'TYR']]
340_KAH	[['477', 'A', 'ALA'], ['478', 'A', 'VAL'], ['479', 'A', 'ALA'], ['480', 'A', 'PRO'], ['482', 'A', 'GLY'], ['483', 'A', 'TYR'], ['484', 'A', 'THR'], ['485', 'A', 'ARG'], ['486', 'A', 'PRO'], ['487', 'A', 'PRO'], ['491', 'A', 'ALA'], ['492', 'A', 'GLY'], ['494', 'A', 'GLU'], ['496', 'A', 'ASP'], ['497', 'A', 'PHE']]

314_KAF	[['463', 'A', 'GLY'], ['464', 'A', 'GLY'], ['465', 'A', 'GLY'], ['466', 'A', 'GLY'], ['467', 'A', 'GLY'], ['468', 'A', 'GLY'], ['474', 'A', 'GLU'], ['475', 'A', 'ALA'], ['476', 'A', 'GLY'], ['477', 'A', 'ALA'], ['478', 'A', 'VAL'], ['479', 'A', 'ALA'], ['480', 'A', 'PRO'], ['481', 'A', 'TYR'], ['482', 'A', 'GLY'], ['483', 'A', 'TYR'], ['484', 'A', 'THR'], ['485', 'A', 'ARG'], ['486', 'A', 'PRO'], ['487', 'A', 'PRO']]
222_KAV	[['171', 'A', 'PHE'], ['172', 'A', 'PRO'], ['173', 'A', 'GLY'], ['174', 'A', 'LEU'], ['175', 'A', 'SER'], ['176', 'A', 'SER'], ['177', 'A', 'CYS'], ['178', 'A', 'SER'], ['179', 'A', 'ALA'], ['183', 'A', 'ASP'], ['184', 'A', 'ILE'], ['185', 'A', 'LEU'], ['186', 'A', 'SER'], ['191', 'A', 'MET'], ['202', 'A', 'VAL'], ['203', 'A', 'SER'], ['213', 'A', 'GLU'], ['214', 'A', 'ALA'], ['215', 'A', 'SER']]
140_KAD	[['163', 'A', 'THR'], ['164', 'A', 'LEU'], ['165', 'A', 'SER'], ['166', 'A', 'LEU'], ['167', 'A', 'LEU'], ['168', 'A', 'GLY'], ['169', 'A', 'PRO'], ['170', 'A', 'THR'], ['171', 'A', 'PHE'], ['172', 'A', 'PRO'], ['175', 'A', 'SER'], ['176', 'A', 'SER'], ['177', 'A', 'CYS'], ['178', 'A', 'SER'], ['180', 'A', 'ASP'], ['181', 'A', 'LEU'], ['182', 'A', 'LYS'], ['185', 'A', 'LEU'], ['192', 'A', 'GLN'], ['193', 'A', 'LEU'], ['194', 'A', 'LEU'], ['195', 'A', 'GLN'], ['196', 'A', 'GLN'], ['197', 'A', 'GLN']]

168_KAK	[['343', 'A', 'SER'], ['344', 'A', 'THR'], ['345', 'A', 'LEU'], ['346', 'A', 'SER'], ['347', 'A', 'LEU'], ['348', 'A', 'TYR'], ['349', 'A', 'LYS'], ['414', 'A', 'LEU'], ['415', 'A', 'HIS'], ['416', 'A', 'GLY'], ['418', 'A', 'GLY'], ['419', 'A', 'ALA'], ['420', 'A', 'ALA'], ['421', 'A', 'GLY'], ['423', 'A', 'GLY'], ['424', 'A', 'SER'], ['426', 'A', 'SER'], ['427', 'A', 'PRO'], ['428', 'A', 'SER'], ['429', 'A', 'ALA'], ['430', 'A', 'ALA'], ['431', 'A', 'ALA'], ['432', 'A', 'SER'], ['433', 'A', 'SER'], ['434', 'A', 'SER'], ['435', 'A', 'TRP'], ['436', 'A', 'HIS'], ['474', 'A', 'GLU'], ['475', 'A', 'ALA'], ['476', 'A', 'GLY'], ['477', 'A', 'ALA'], ['478', 'A', 'VAL'], ['479', 'A', 'ALA'], ['480', 'A', 'PRO'], ['481', 'A', 'TYR'], ['482', 'A', 'GLY'], ['484', 'A', 'THR'], ['485', 'A', 'ARG'], ['486', 'A', 'PRO'], ['490', 'A', 'LEU'], ['491', 'A', 'ALA'], ['492', 'A', 'GLY'], ['493', 'A', 'GLN']]
22_KAI	[['492', 'A', 'GLY'], ['493', 'A', 'GLN'], ['502', 'A', 'VAL'], ['503', 'A', 'TRP'], ['504', 'A', 'TYR'], ['509', 'A', 'VAL'], ['510', 'A', 'SER'], ['511', 'A', 'ARG'], ['512', 'A', 'VAL'], ['513', 'A', 'PRO'], ['514', 'A', 'TYR'], ['515', 'A', 'PRO'], ['516', 'A', 'SER'], ['519', 'A', 'CYS'], ['520', 'A', 'VAL'], ['521', 'A', 'LYS'], ['522', 'A', 'SER'], ['523', 'A', 'GLU'], ['524', 'A', 'MET'], ['528', 'A', 'MET'], ['529', 'A', 'ASP']]

156_KAE	[['171', 'A', 'PHE'], ['172', 'A', 'PRO'], ['173', 'A', 'GLY'], ['175', 'A', 'SER'], ['176', 'A', 'SER'], ['177', 'A', 'CYS'], ['178', 'A', 'SER'], ['180', 'A', 'ASP'], ['181', 'A', 'LEU'], ['182', 'A', 'LYS'], ['183', 'A', 'ASP'], ['184', 'A', 'ILE'], ['185', 'A', 'LEU'], ['186', 'A', 'SER'], ['187', 'A', 'GLU'], ['188', 'A', 'ALA'], ['189', 'A', 'SER'], ['191', 'A', 'MET'], ['192', 'A', 'GLN'], ['193', 'A', 'LEU'], ['195', 'A', 'GLN']]
216_KAA	[['466', 'A', 'GLY'], ['467', 'A', 'GLY'], ['468', 'A', 'GLY'], ['469', 'A', 'GLY'], ['474', 'A', 'GLU'], ['475', 'A', 'ALA'], ['476', 'A', 'GLY'], ['477', 'A', 'ALA'], ['478', 'A', 'VAL'], ['483', 'A', 'TYR'], ['484', 'A', 'THR'], ['485', 'A', 'ARG'], ['487', 'A', 'PRO'], ['488', 'A', 'GLN'], ['489', 'A', 'GLY'], ['490', 'A', 'LEU'], ['491', 'A', 'ALA'], ['492', 'A', 'GLY'], ['493', 'A', 'GLN'], ['494', 'A', 'GLU'], ['495', 'A', 'SER'], ['496', 'A', 'ASP'], ['497', 'A', 'PHE'], ['498', 'A', 'THR'], ['499', 'A', 'ALA'], ['501', 'A', 'ASP'], ['502', 'A', 'VAL'], ['503', 'A', 'TRP'], ['504', 'A', 'TYR'], ['506', 'A', 'GLY'], ['508', 'A', 'MET'], ['509', 'A', 'VAL'], ['511', 'A', 'ARG']]

Table S2: Residues involved in the 41 selected binding sites of AR-V7

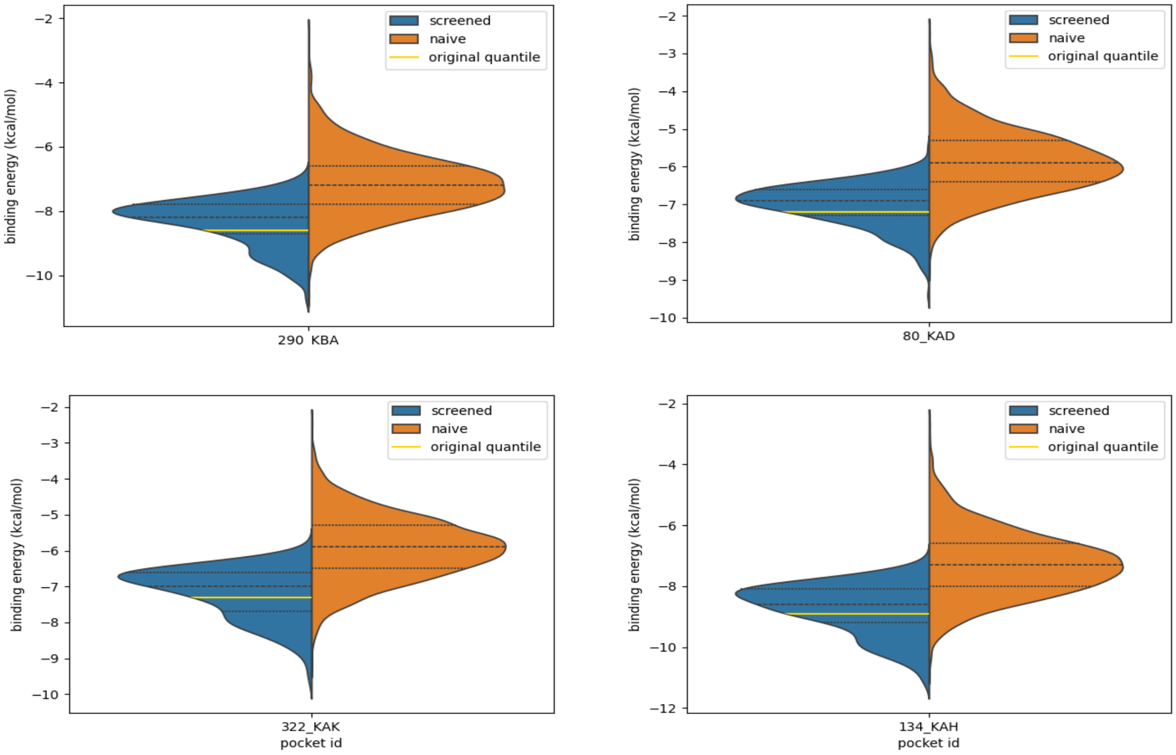

Figure S8: Distribution of binding energies for screened (in blue) and naive docked molecules (orange) compared and 'activity' binding site threshold (yellow).

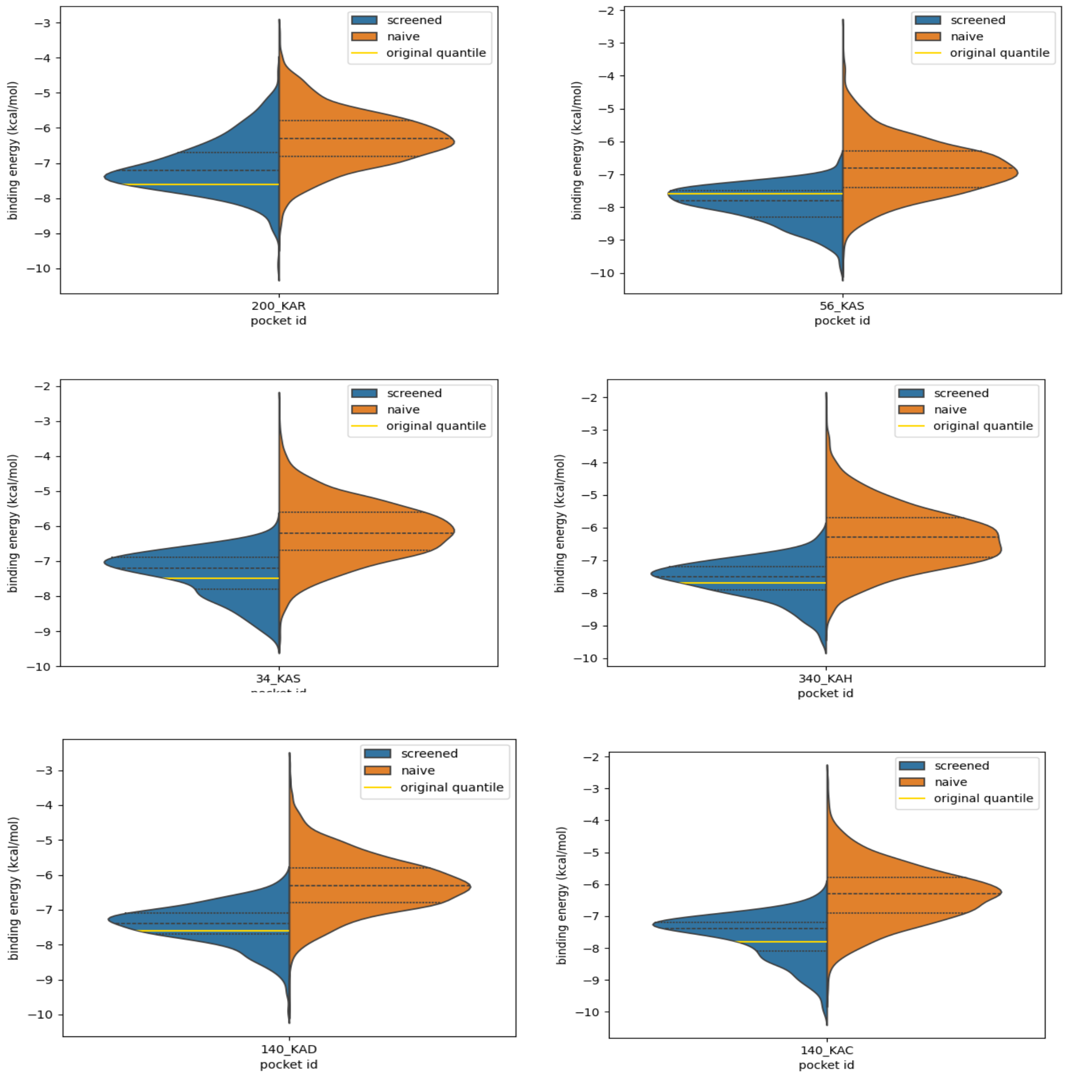

Figure S9: Distribution of binding energies for screened (in blue) and naive docked molecules (orange) compared and 'activity' binding site threshold (yellow).

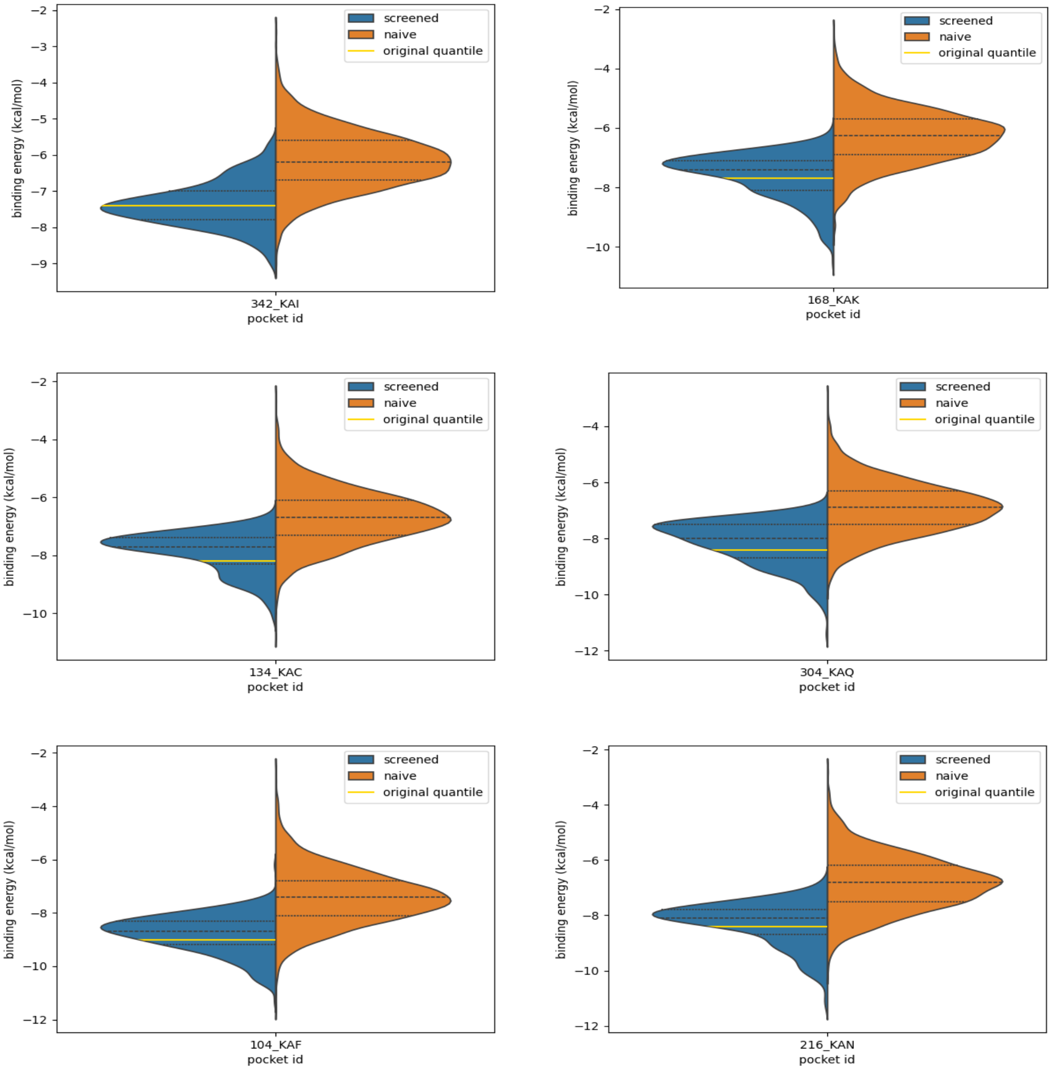

Figure S10: Distribution of binding energies for screened (in blue) and naive docked molecules (orange) compared and 'activity' binding site threshold (yellow).

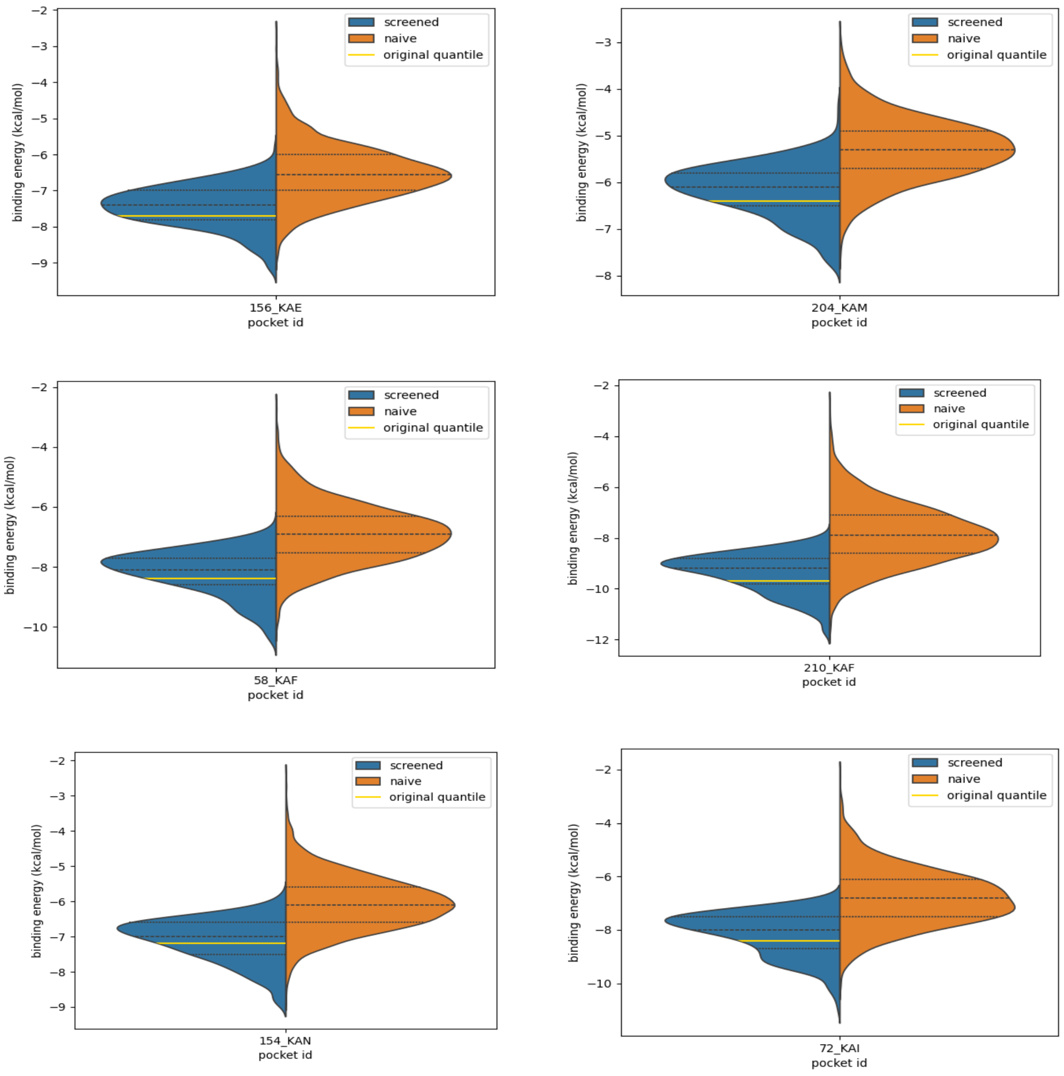

Figure S11: Distribution of binding energies for screened (in blue) and naive docked molecules (orange) compared and 'activity' binding site threshold (yellow).

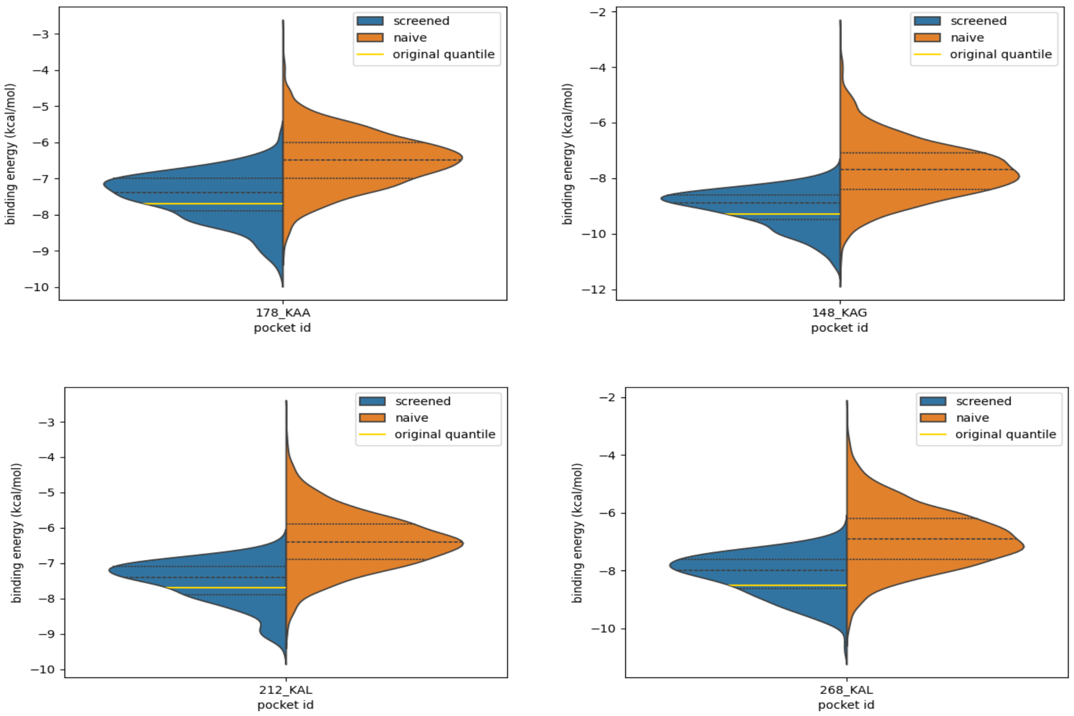

Figure S12: Distribution of binding energies for screened (in blue) and naive docked molecules (orange) compared and 'activity' binding site threshold (yellow).

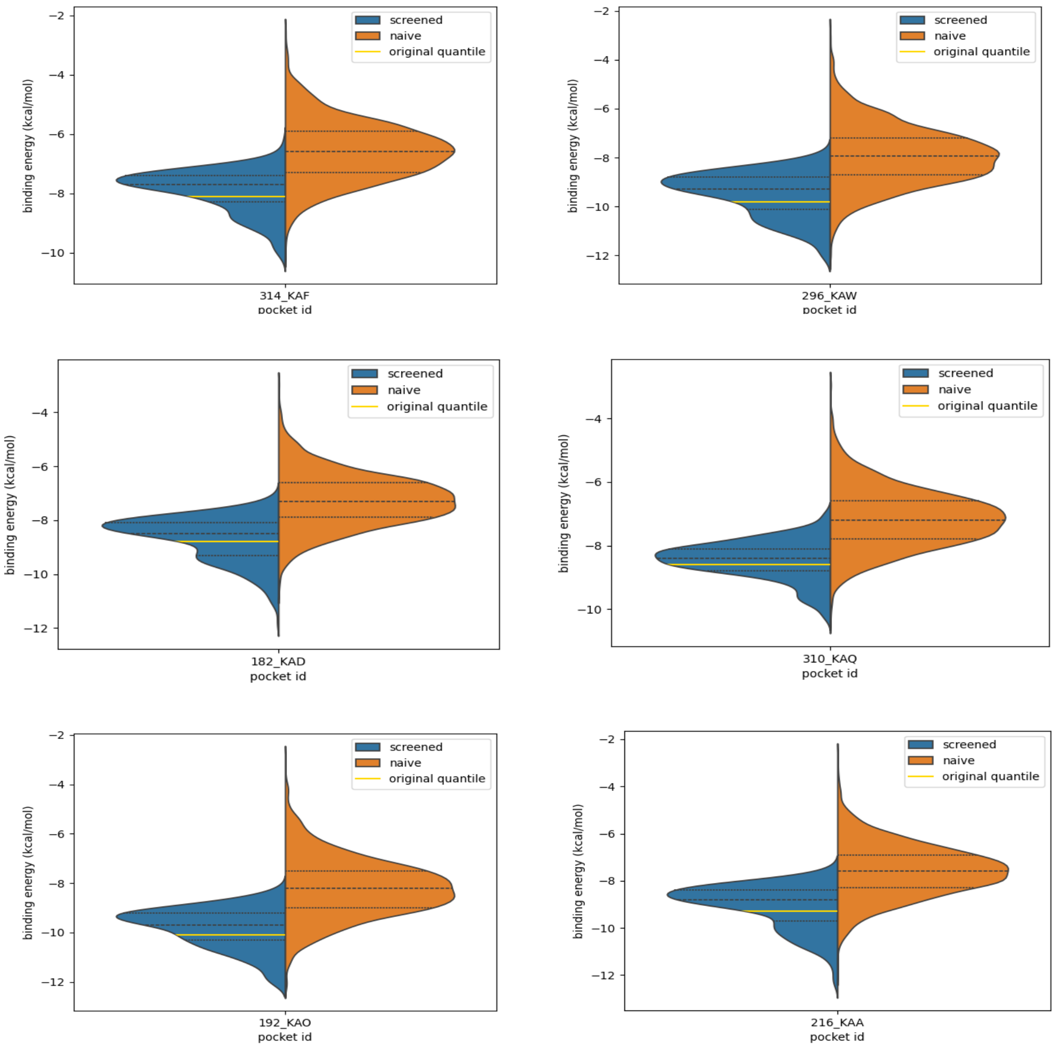

Figure S13: Distribution of binding energies for screened (in blue) and naive docked molecules (orange) compared and 'activity' binding site threshold (yellow).

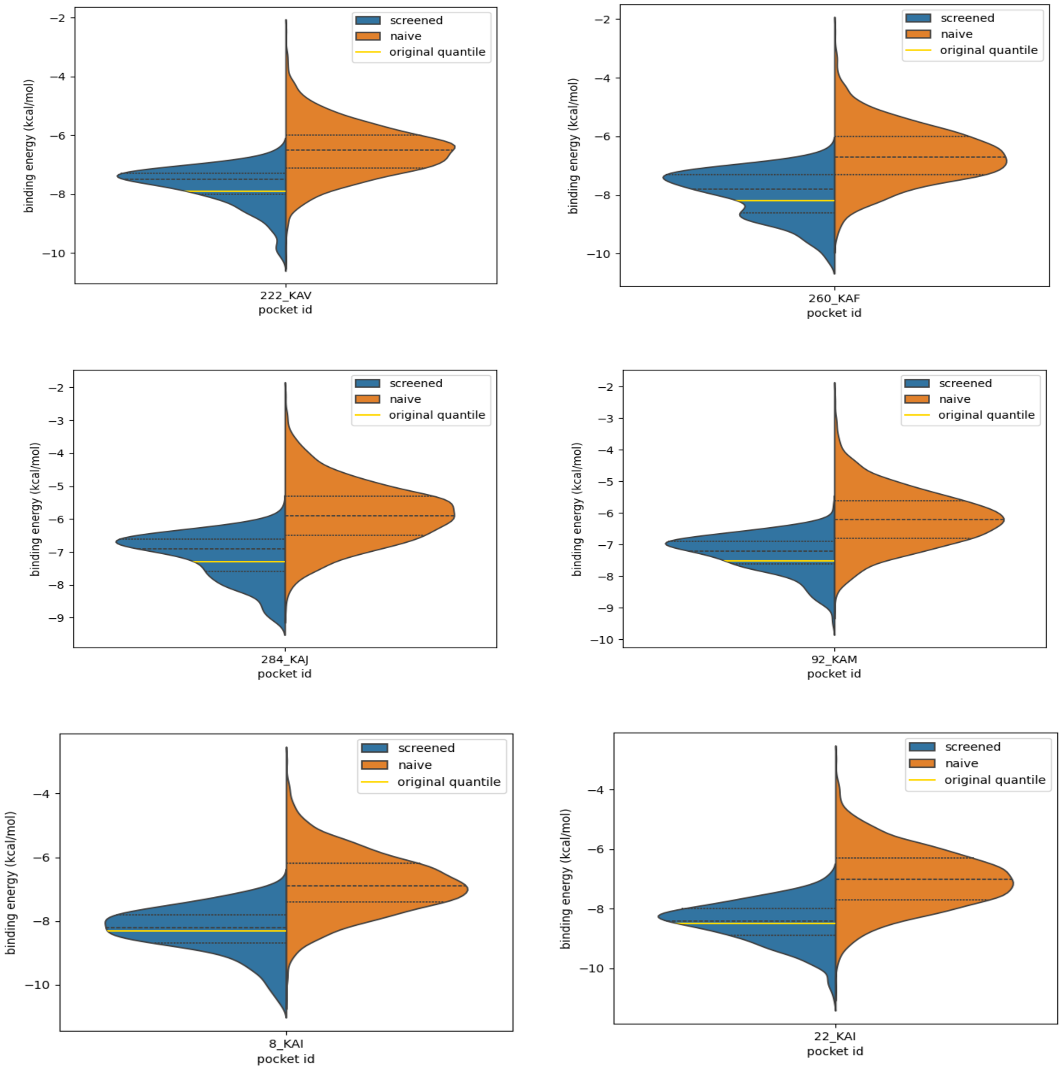

Figure S14: Distribution of binding energies for screened (in blue) and naive docked molecules (orange) compared and 'activity' binding site threshold (yellow).

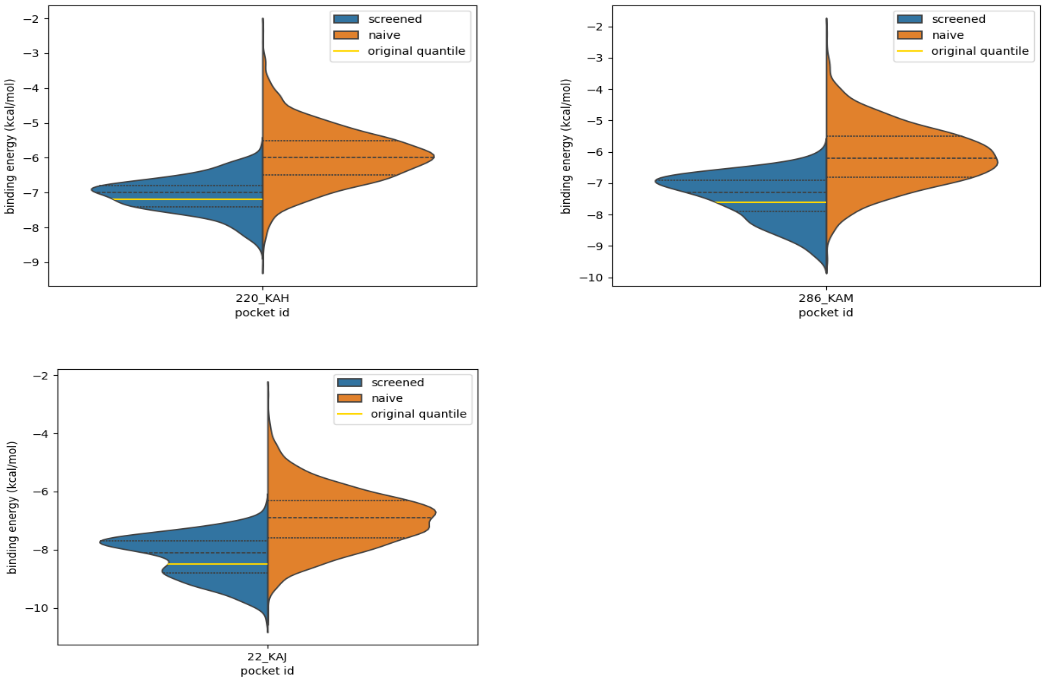

Figure S15: Distribution of binding energies for screened (in blue) and naive docked molecules (orange) compared and 'activity' binding site threshold (yellow).

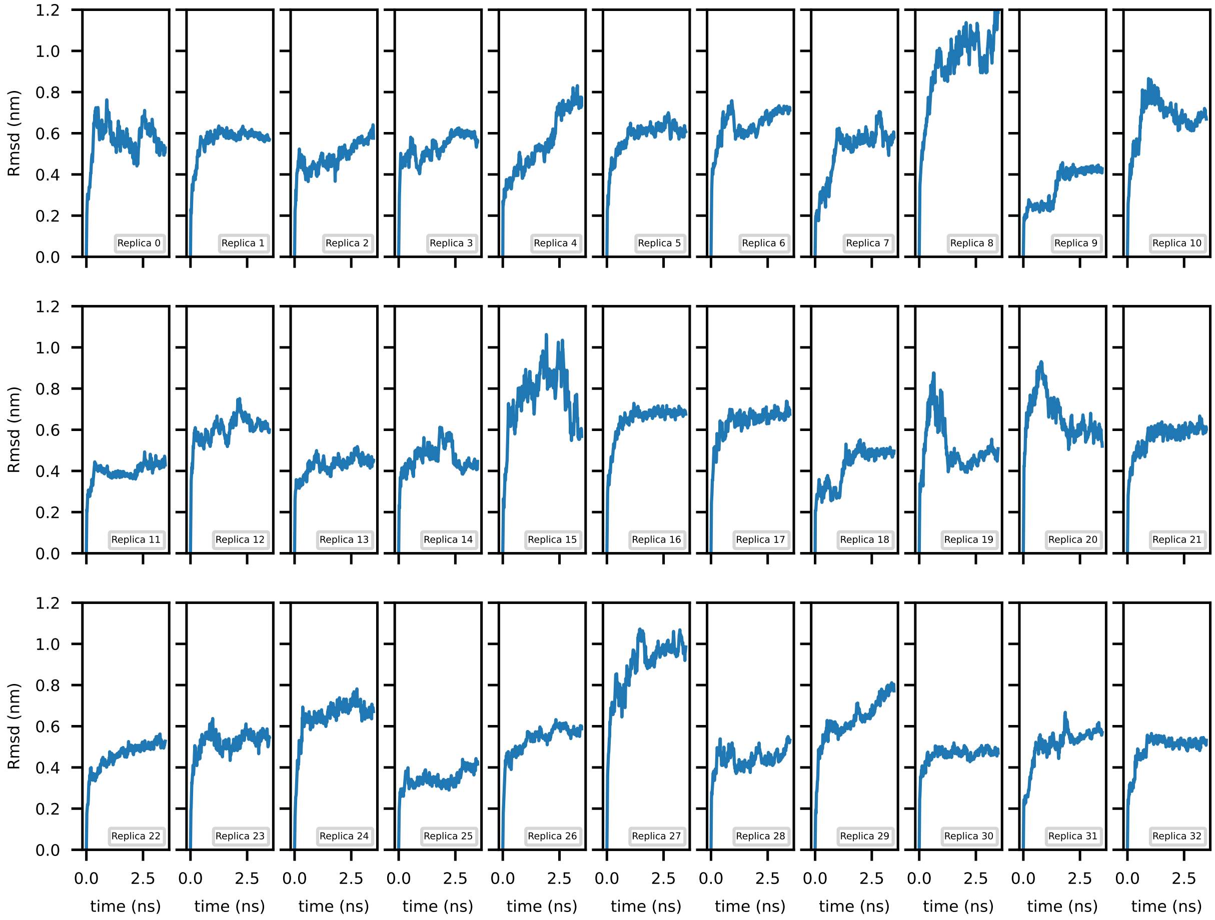

Figure S16: Pose stability. Root Mean Square deviation of pocket forming residues and ChEMBL22003 along the atomistic PB-MetaD with respect to the starting configuration originating from the AlphaFold Metainference for the protein and VINA docking for the compound. Various subplots signify different replicas where the compound is bound to different binding sites, 33 in total.
